# Exome variant prioritization in a large cohort of hearing-impaired individuals indicates *IKZF2* to be associated with non-syndromic hearing loss and guides future research of unsolved cases

**DOI:** 10.1007/s00439-024-02706-w

**Published:** 2024-10-16

**Authors:** Hedwig M. Velde, Maryam Vaseghi-Shanjani, Jeroen J. Smits, Gayatri Ramakrishnan, Jaap Oostrik, Mieke Wesdorp, Galuh Astuti, Helger G. Yntema, Lies Hoefsloot, Cris P. Lanting, Martijn A. Huynen, Anna Lehman, Stuart E. Turvey, DOOFNL Consortium, E. Aten, M. J. van den Boogaard, F. L. J. Cals, M. F. van Dooren, F. A. Ebbens, I. Feenstra, R. H. Free, H. H. W. de Gier, T. P. M. Goderie, L. Haer-Wigman, K. Hellingman, E. H. Hoefsloot, J. R. Hof, J. van de Kamp, S. G. Kant, J. S. Klein Wassink-Ruiter, H. Kremer, M. Kriek, A. M. A. Lachmeijer, C. P. Lanting, S. M. Maas, P. Merkus, R. J. E. Pennings, A. Plomp, F. G. Ropers, L. J. C. Rotteveel, M. P. van der Schroeff, A. L. Smit, J. J. Smits, V. Vernimmen, J. C. C. Widdershoven, H. G. Yntema, Ronald J. E. Pennings, Hannie Kremer

**Affiliations:** 1 Department of Otorhinolaryngology, Radboudumc, Nijmegen, The Netherlands; 2 Donders Institute for Brain, Cognition and Behaviour, Radboudumc, Nijmegen, The Netherlands; 3 Department of Pediatrics, The University of British Columbia and BC Children’s Hospital, Vancouver, BC Canada; 4 Department of Clinical Genetics, University Medical Center Utrecht, Utrecht, The Netherlands; 5 Department of Medical Biosciences, Radboudumc, Nijmegen, The Netherlands; 6 Department of Human Genetics, Radboudumc, Nijmegen, The Netherlands; 7 Department of Clinical Genetics, Erasmus Medical Center, Rotterdam, The Netherlands; 8 Center for Molecular and Biomolecular Informatics, Radboudumc, Nijmegen, The Netherlands

## Abstract

Although more than 140 genes have been associated with non-syndromic hereditary hearing loss (HL), at least half of the cases remain unexplained in medical genetic testing. One reason is that pathogenic variants are located in ‘novel’ deafness genes. A variant prioritization approach was used to identify novel (candidate) genes for HL. Exome-wide sequencing data were assessed for subjects with presumed hereditary HL that remained unexplained in medical genetic testing by gene-panel analysis. Cases in group AD had presumed autosomal dominantly inherited HL (n = 124), and in group AR, presumed autosomal recessive HL (n = 337). Variants in known and candidate deafness genes were prioritized based on allele frequencies and predicted effects. Selected variants were tested for their co-segregation with HL. Two cases were solved by variants in recently identified deafness genes (*ABHD12*, *TRRAP*). Variant prioritization also revealed potentially causative variants in candidate genes associated with recessive and X-linked HL. Importantly, missense variants in *IKZF2* were found to co-segregate with dominantly inherited non-syndromic HL in three families. These variants specifically affected Zn^2+^-coordinating cysteine or histidine residues of the zinc finger motifs 2 and 3 of the encoded protein Helios. This finding indicates a complex genotype–phenotype correlation for *IKZF2* defects, as this gene was previously associated with non-syndromic dysfunction of the immune system and ICHAD syndrome, including HL. The designed strategy for variant prioritization revealed that *IKZF2* variants can underlie non-syndromic HL. The large number of candidate genes for HL and variants therein stress the importance of inclusion of family members for variant prioritization.

**Supplementary Information:**

The online version contains supplementary material available at 10.1007/s00439-024-02706-w.

## Introduction

Hereditary hearing loss (HHL) has been the subject of extensive research for decades. In 1950, Kinney asserted that the causes of HHL were still far from being unveiled (Kinney 1950). At that time, the likelihood of multiple forms of HHL had already been established (Keizer 1952). Until now, our knowledge has expanded to more than 140 genes and over 9000 (likely) pathogenic variants being associated with non-syndromic hearing loss (HL) (Van Camp and Smith) and a larger number of genes for syndromic HL (Booth and Shearer 2022; McKusick-Nathans Institute of Genetic Medicine n.d.). Despite the significant progress made, the diagnostic yield of genetic analysis through targeted sequencing or exome sequencing targeting a hearing-loss gene panel remains between 25 and 50% (Perry et al. 2023; Sloan-Heggen et al. 2016; Tropitzsch et al. 2022; Zazo Seco et al. 2017). Therefore, a comprehensive insight into the causes of HHL is yet to be attained despite the accomplishments in understanding HHL since Kinney’s words.

Knowledge of the specific genetic causes of HL is of great importance. It facilitates accurate prognostic and genetic counselling. Understanding the anticipated progression and eventual degree of HL, as well as the potential manifestation of additional symptoms in the case of a syndromic diagnosis, can be vital for therapeutic management. Also, the identification of the mode of inheritance allows for informed family planning decisions. Importantly, unravelling the hidden genetic landscape of HHL is critical for the development and evaluation of (genetic) therapies as is exemplified by the recent study on gene augmentation therapy for *OTOF*-associated HL in humans (Lv et al. 2024).

The reasons for missing genetic diagnoses in diagnostic genetic testing have been extensively discussed (Kremer 2019, 2022). These include limitations of the DNA-sequencing methods and interpretation of identified variants (de Bruijn et al. 2021, 2023). In the present study, we focused on identifying genes that had not yet been associated with HL in humans in a large cohort of subjects with presumed HHL. Clinical exome sequencing and targeted analysis of a deafness gene panel did not unveil the cause of HL in these subjects. Using a variant prioritization approach, a number of potential causative variants in candidate genes for recessively inherited and X-linked HL were identified, and two index cases could be explained by variants in recently identified genes for HL. Importantly, we also found that *IKZF2* is associated with autosomal dominantly inherited HL.

## Material and methods

### Subject identification

Subjects were recruited from seven academic medical centers in the Netherlands, that collaborate in the DOOFNL consortium: Amsterdam UMC, Erasmus Medical Center, Leiden University Medical Center, Maastricht UMC+, Radboud University Medical Center (Radboudumc, coordinating center), University Medical Center Groningen and UMC Utrecht. Patients were selected for inclusion by their otolaryngologist or clinical geneticist. Subjects were eligible for inclusion if their HL was sensorineural, presumably of genetic origin, and if the most recent gene panel for HL (addressed by exome sequencing), at the time of testing, did not result in a (likely) genetic diagnosis (https://www.radboudumc.nl/en/patient-care/patient-examinations/exome-sequencing-diagnostics/exomepanelspreviousversions/exomepanelspreviousversions/hearing-impairment, https://www.erasmusmc.nl/nl-nl/patientenzorg/laboratoriumspecialismen/klinische-genetica#35d085e6-2dc0-48a3-9dfd-8aa502ca959e). Patients with an onset of HL after the age of 50 and/or a presumably syndromic form of HL were excluded.

### Variant extraction from exome sequencing data

Clinical exome sequencing was performed either in the ISO 15189 accredited Genome Diagnostic Laboratory of the department of Human Genetics at Radboudumc (Haer-Wigman et al. 2017; Velde et al. 2023) or in the ISO 15189 accredited Diagnostic Laboratory of the department of Clinical Genetics at Erasmus MC (Haarman et al. 2022). Initial clinical exome sequencing analysis for included subjects was conducted from 2013 to 2020. In that period, the following enrichment kits were used: Agilent SureSelectXT Human All Exon 50 Mb Kit (Agilent-V4), Agilent SureSelectQXT Human All Exon V5 Kit (Agilent-V5) and Agilent SureSelect Human All Exon V7 Kit (Agilent-V7). For the present study, Variant Call Format (VCF) files were re-annotated using an in-house bioinformatics pipeline (Schobers et al. 2022). As requested by the medical ethics committee, variants in genes that could lead to secondary findings, as indicated by the American College of Medical Genetics and Genomics (ACMG) based on the medical actionability of the associated conditions, were excluded before further analysis in this study (Miller et al. 2021). All other variants were analysed.

### Variant prioritization

**Fig. 1:**
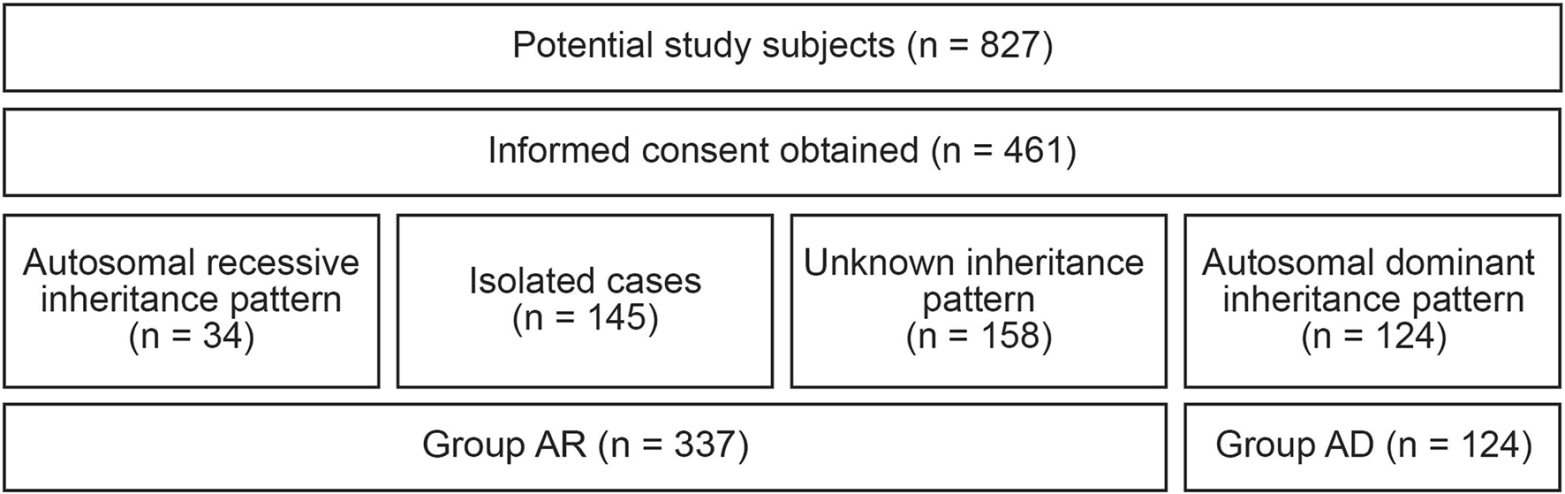
Flowchart of subject inclusion and group allocation. AD, autosomal dominant; AR, autosomal recessive

Two groups were defined for further analyses (Fig. 1). Group AR consisted of index cases with a presumed autosomal recessive inheritance pattern of HL, individuals with an unknown inheritance pattern, and isolated cases. Group AD consisted of index cases with a presumed autosomal dominant inheritance pattern of HL. None of the participating subjects were indicated to suffer from X-linked HL.

*As a first step*, variants were excluded based on allele frequencies (AF) in the Genome Aggregation Database [gnomAD, v2.1.1 (Karczewski et al. 2020)] and an in-house database. In group AR, the criterion for exclusion was an AF ≥ 1% in either database; in group AD, this was an AF ≥ 0.1%.

*In the second step*, the remaining variants were prioritized based on their predicted effects on the encoded proteins. All variants introducing a stop gain or loss, frameshift variants, (non-)canonical splice site variants (non-canonical splice acceptor variants: positions − 3 to − 20; non-canonical splice donor variants: positions + 3 to + 8), in-frame deletions or insertions and variants with a predicted effect on splicing by SpliceAI (threshold: ≥ 0.2/1) (Jaganathan et al. 2019) were included. Missense variants were included if at least one of three in silico tools predicted the variant to be deleterious. Prediction tools and thresholds used in this phase were PhyloP (≥ 2.7) (Pollard et al. 2010), Combined Annotation Dependent Depletion (CADD_PHRED; ≥ 15) (Kircher et al. 2014) and Grantham Score (≥ 80) (Grantham 1974). These tools were incorporated into the in-house bioinformatics pipeline.

*As a third step*, included variants were prioritized based on internal gene lists of either known human deafness genes or candidate genes (Supplemental Table 1). Four (composite) gene lists were used: (1) human deafness genes [composed of known non-syndromic and syndromic deafness genes based on the Hereditary Hearing Loss Homepage (Van Camp and Smith), literature and OMIM (McKusick-Nathans Institute of Genetic Medicine n.d.)], (2) mouse deafness genes (based on literature, the Mouse Genome Informatics Website (Baldarelli et al. 2021; Blake et al. 2021; Krupke et al. 2017) and personal communication), (3) genes with preferential inner ear expression (Luijendijk et al. 2003; Robertson et al. 1994; Schrauwen et al. 2016) and (4) other candidate genes (based on literature and personal communication). In the AR group, variants in twelve genes with a disproportionately large number of variants were excluded *(AHNAK2*, *ANKRD36*, *ANKRD36C*, *CDC27*, *CTBP2*, *DNAH7*, *FAM86B1*, *MUC21*, *MUC22*, *MUC6*, *NACA* and *RRBP1)*. These were genes from the list of genes with preferential inner ear expression (Supplemental Table 1, list 3).

**Table 1:** Follow-up of selected variants in human deafness genes in group AR

Gene	Variant	Findings^a^
*ABHD12*	Chr20(GRCh37):g.25282937del NM_001042472.3:c.1075del p.(Val359Phefs*27) (homozygous)	Likely cause of HL, no segregation analysis possible Gene not yet in HL gene panel for clinical exome sequencing
*LRP2*	Chr2(GRCh37):g.170002322G > A NM_004525.3:c.12923C > T p.(Thr4308Met)	Variants do not co-segregate with HL
	Chr2(GRCh37):g.170070387G > T NM_004525.3:c.5827-7C > A p.?	
	Chr2(GRCh37):g.170081863G > T NM_004525.3:c.5495C > A p.(Ser1832*)	
	Chr2(GRCh37):g.170081873C > A NM_004525.3:c.5485G > T p.(Asp1829Tyr)	

Variants are in heterozygous state unless stated otherwise

^a^One cell per subject

For the subsequent step, candidate variants were evaluated separately for the AR and AD groups. In group AR, we selected genes with ≥ 2 variants and thus potentially biallelic variants. We excluded variants in candidate genes (lists 2, 3 and 4) linked to disease in OMIM because pathogenic variants in those genes were more likely to be associated with other conditions, whereas our cohort consisted of individuals with non-syndromic HL. Copy number variants (CNVs) were addressed for genes in specific cases with a strong, *i.e.*, truncating or canonical splice site, monoallelic variant in a known human deafness gene (list 1) or candidate deafness gene (lists 2 and 3). The presence of CNVs was assessed by Copy Number Inference From Exome Reads (CoNIFER (Krumm et al. 2012)) or ExomeDepth (Plagnol et al. 2012), depending on which tool was employed upon clinical exome sequencing for the relevant DNA samples. CNVs were only followed up if the gene was not known to be highly variable in the general population (excluded genes were *MUC17* and *OR2A7*) (Lewis et al. 2022). Genomic qPCR was performed according to standard protocols for putative CNVs to confirm their presence.

In group AD, variants in genes known to be associated with dominantly inherited HL were evaluated as well as variants in orthologs of mouse deafness genes (list 2). To facilitate variant interpretation and follow-up in this group, we focused on variants present in at least three cases. To limit the number of variants to an amount feasible for follow-up, a subgroup of mouse deafness genes (list 2) was used in group AD, based on personal communication and literature (Supplemental Table 1).

In both the AR and AD groups, an additional filtering step was performed for missense variants. Four in silico tools were used with the indicated thresholds: Sorting Intolerant From Tolerant (SIFT; ≤ 0.05) (Ng and Henikoff 2001), Polymorphism Phenotyping v2 (PolyPhen-2; ≥ 0.450) (Adzhubei et al. 2010) and MutationTaster (deleterious) (Schwarz et al. 2014) and again CADD_PHRED (≥ 15). If three or all four of these tools predicted a variant to be non-pathogenic, the variant was excluded. Alignment files (BAM or CRAM, depending on which was available for the relevant DNA samples) were assessed to exclude variants likely to be calling artefacts. In group AR, these files were also used to determine whether variants in close proximity to each other affected the same allele or not.

### Variant follow-up

For variants that passed the prioritization steps, clinical files of the relevant cases were reviewed to determine whether a variant had already been evaluated as not causative in clinical exome sequencing. This review also aimed to confirm the presumed inheritance pattern. For variants in known human deafness genes, we also compared the subject’s phenotype with the phenotype associated with the relevant deafness gene. Co-segregation with HL in the corresponding families was addressed for variants that passed these steps. Variants were validated by PCR and Sanger sequencing according to standard protocols.

As many family members as necessary and feasible were included to either exclude or confirm co-segregation of the variant with HL. In this process, we considered ethical implications for potentially pre-symptomatic individuals in case of late-onset HL.

Likely causative variants in known human deafness genes, as well as *IKZF2* variants, were classified using the ACMG criteria and its specifications for HL (Oza et al. 2018; Richards et al. 2015).

### Characterization of *IKZF2*-associated HL

To characterize the hearing phenotype, we retrospectively collected audiometric data. Subjects were considered affected when thresholds higher than the age- and sex-specific 95th percentile (International Organization for Standardization, ISO 7029:2017 (International Organization for Stardardization 2017)) were observed in pure tone audiometry for at least three frequencies of the best hearing ear. HL was considered asymmetric if a difference of at least 10 decibel hearing level (dB HL) was observed between both ears at two or more frequencies (Gendeaf Study Group et al. 2003). The pure tone average was defined as the mean of pure tone thresholds at 0.5, 1, 2 and 4 kHz (PTA_0.5–4 kHz_). The PTA of the best-hearing ear (better ear PTA) was used for the analyses. Age-related typical audiograms (ARTA) were derived from linear regression analysis of all audiometric data according to previously described methods (Huygen et al. 2003). The right ear of subject III:1 from family W22-1907 was excluded because the HL in that ear was considered unrelated to the *IKZF2* variant. In addition, all affected study participants with an *IKZF2* variant were requested to complete a questionnaire on audiovestibular function and general medical history. This questionnaire included the dizziness handicap inventory (DHI), consisting of an emotional (E, maximum score of 36), functional (F, maximum score of 36) and physical (P, maximum score of 28) subdomain (Jacobson and Newman 1990; Whitney et al. 2004). If possible, and with permission of the relevant subject, missing data were retrieved from medical records.

### VNTR marker analysis

Haplotypes in the *IKZF2* chromosomal region were determined by genotyping variable number of tandem repeat (VNTR) markers as described (Wesdorp et al. 2018).

### Protein structural analysis

Missense variants in *IKZF2* in two C2H2 domains of the encoded zinc finger (ZF) protein Helios were addressed: Chr2(GRCh37):g.213914526T > G NM_001387220.1:c.485A > C NP_001374149.1:p.(His162Pro) and Chr2(GRCh37):g.213914577C > A NM_001387220.1:c.434G > T NP_001374149.1:p.(Cys145Phe) at the second N-terminal C2H2 domain; and Chr2(GRCh37):g.213914502C > T NM_001387220.1:c.509G > A NP_001374149.1:p.(Cys170Tyr) at the third N-terminal C2H2 domain. Based on the availability of protein structural data, the impact of missense variants in the second C2H2 domain was investigated using its crystal structure (PDB: 7LPS). For the variant in the third C2H2 domain, reliable structural models from AlphaFold were obtained using default parameters (Jumper et al. 2021; Varadi et al. 2022). To predict and understand the functional outcomes of the missense variants we estimated changes in protein stability brought about by the variants using the FoldX energy function (version 5.0) (Schymkowitz et al. 2005). We first constructed structural models for each missense variant using the RepairPDB and BuildModel functions with five iterations of rotamer adjustments in the amino acid side chains. Subsequently, we calculated the mean differences in protein stability (free energy) between the variant and wild-type (WT) structures, expressed in kcal/mol. A mean difference (∆G_mut_ − ∆G_wt_) of > 1 kcal/mol was classified as destabilizing.

### Functional evaluation of *IKZF2* variants

Assessment of Helios protein expression and luciferase reporter assays to functionally validate the effect of the three novel *IKZF2* variants were performed as described (Mohajeri et al. 2023) and summarized below. The collaboration between Radboudumc and The University of British Columbia in this study was initiated via GeneMatcher (Sobreira et al. 2015).

Helios expression was assessed by immunoblotting upon transfection of Human Embryonic Kidney (HEK293) cells with 3 μg pCMV6-XL4 plasmids encoding FLAG-tagged WT or variant Helios using Lipofectamine™ 3000 Transfection Reagent (Thermo Fisher Scientific). When co-transfection of WT and variant plasmids was performed, a total amount of 3 μg plasmids were employed. Whole cell lysates were separated by 10% SDS-PAGE and transferred onto polyvinylidene difluoride membranes (MilliporeSigma). Prior to SDS-PAGE, protein concentrations were measured using Pierce™ Coomassie Plus (Bradford) Assay Reagent. For the detection of FLAG-tagged Helios and β-Actin the employed primary antibodies were the following: anti-Helios, Cell Signaling Technology, cat# 89270, 1:1000, anti-FLAG, Sigma Aldrich, cat# F1804, 1:1000 and anti-β-Actin, Cell Signaling Technology, cat# 3700, 1:20,000. As secondary antibodies, anti-rabbit (IgG DyLight 800, Rockland Immunochemicals) and anti-mouse (IgG IRDye 680RD, LI-COR) were used at a concentration of 1:20,000. The membranes were imaged using a LI-COR Odyssey infrared scanner (LI-COR Biosciences). The bands were quantified using Image Studio™ Lite (LI-COR) and normalized to the corresponding β-Actin expression levels.

For luciferase reporter assays, the − 580 to + 57 region of the human *IL2* promoter was cloned into the PGL4.14 (Promega) firefly luciferase reporter plasmid. Using Lipofectamine 3000 Transfection (Thermo Fisher Scientific), HEK293 cells were co-transfected with the *IL2* promotor containing luciferase reporter plasmid, a PGL4.74 (Promega) Renilla luciferase control plasmid and the aforementioned plasmids encoding no (empty vector (EV)), WT or each of the variant Helios. In addition, co-transfections were conducted where cells received a combination of WT and each of the variant *IKZF2* plasmids in increasing ratios of variant to WT (0.25:1, 0.5:1, 1:1, 2:1 and 4:1) totalling to 250 ng. Additionally, all cells were also transfected with 250 ng of *IL2*-luciferase reporter plasmid and 10 ng of PGL4.74 Renilla luciferase control plasmid (Promega). Cells were lysed by adding 1 × Glo Lysis Buffer (Promega) and transferred to white flat-bottom 96-well plates in technical triplicates. The Dual-Glo Luciferase Assay Kit (Promega) was used according to manufacturer’s recommendations and luciferase activity was measured using the Infinite M200 plate reader (Tecan). In the analysis step, the firefly luciferase activity was normalized against Renilla luciferase. The normalized firefly luciferase activity was further divided by the normalized value from the EV condition.

All data are presented as mean ± standard error of the mean (SEM). Statistical analysis of Helios quantification on immunoblot was conducted using a parametric one-way ANOVA. This analysis was specifically designed to compare each variant group individually to the WT group. Each row of data represented matched measures, corresponding to results from independently conducted experiments. It is important to note that we did not perform corrections for multiple comparisons in this analysis in order to focus exclusively on the pairwise comparison of each variant group with the WT group, without considering the influence of other groups. This approach allowed for a direct and isolated assessment of the differences between each variant and the WT protein expression levels. Statistical analysis of data derived from the luciferase reporter assays was conducted using a parametric one-way ANOVA, using Dunnett's test to account for multiple comparisons. Statistical significance is presented as follows: *p*-val < 0.05 (*), *p*-val < 0.01 (**), *p*-val < 0.001 (***) and *p*-val < 0.0001 (****).

## Results

### Subject identification and general steps of variant prioritization

A total of 827 subjects were identified as potential study participants and they or their legal representatives were informed about the study (Fig. 1). Written informed consent was obtained from 461 subjects. With only a few exceptions, the individuals enrolled in this study were index cases. For analysis of exome sequencing data, 34 subjects with presumed autosomal recessive inheritance of HL were combined with 145 isolated cases with HL and 158 cases with HL with an unknown inheritance pattern (group AR; n = 337). Based on family history, 124 subjects had HL with a presumed autosomal dominant inheritance pattern (group AD).

After AF-based filtering of variants, 900,417 variants remained in group AR and 200,531 in group AD. The average numbers of variants per sample were 2,672 and 1,617 in group AR and AD, respectively. Subsequent filtering of variants using prediction tools yielded 61,180 variants in group AR (average per sample: 182) and 27,089 variants in group AD (average per sample: 218).

### Variant prioritization in group AR

#### Known human deafness genes

In group AR, 2247 variants were identified in known human deafness genes (Supplemental Table 1, list 1; Supplemental Table 2). Variants in samples without a second variant in the same gene were excluded, after which 315 variants remained. Hemizygous variants on the X chromosome were included. For follow-up feasibility, the most promising variants were targeted through an additional filtering step, specifically selecting samples in which at least one of the variants was predicted to have a truncating effect. This revealed 102 variants, of which 92 were excluded after manual assessment of alignment files. Most of the excluded variants were found in older files or involved contiguous nucleotides. Of the remaining ten variants, four were excluded because the phenotype of the subjects did not match the phenotype reported in the literature, and one was excluded because the case had already been solved (Chen et al. 2021) but was not correctly removed from the cohort of unsolved cases (Supplemental Table 3). Five variants, of which one in homozygous state, were selected for follow-up (Table 1).A homozygous truncating *ABHD12* variant (ACMG classification ‘pathogenic’: PVS1, PM2, PM3_supp, PP3, PP4) was the likely cause of HL in the subject. *ABHD12* is associated with a combination of HL, polyneuropathy, ataxia, retinitis pigmentosa and cataracts (OMIM 612674). Upon review of the subject’s clinical records, the presence of cataracts in addition to moderate-to-severe HL since childhood (Supplemental Fig. 2A) was noted. Besides the *ABHD12* variant, four *LRP2* variants were selected for follow-up. These four variants were identified in one subject and did not co-segregate with HL in the family.

**Table 2:** Possibly causative variants in candidate deafness genes in group AR

Gene	Variant	Findings^a^
*ANKRD17*	Chr4(GRCh37):g.74027206T > C NM_032217.5:c.548-141A > G p.? (homozygous)	Possibly causative, no segregation analysis possible
*ELK1*	ChrX(GRCh37):g.47497233G > A NM_001114123.3:c.1003C > T p.(Arg335Trp) (hemizygous)	Possibly causative, no segregation analysis possible
*ENOX2*	ChrX(GRCh37):g.129822965G > A NM_182314.3:c.212C > T p.(Thr71Ile) (hemizygous)	Possibly causative, no segregation analysis possible
*HEPH*	ChrX(GRCh37):g.65427064G > T NM_138737.4:c.2481G > T p.(Lys827Asn) (hemizygous)	Possibly causative, no further segregation analysis possible
*SHPRH*	Chr6(GRCh37):g.146244887A > G NM_001370327.1:c.3437 T > C p.(Ile1146Thr)	Possibly causative, no further segregation analysis possible
	Chr6(GRCh37):g.146271582 T > G NM_001042683.1:c.800A > C p.(Glu267Ala)	
*SRPX*	ChrX(GRCh37):g.38009034A > G NM_006307.5:c.1325 T > C p.(Ile442Thr) (hemizygous)	Possibly causative, no further segregation analysis possible
*YIPF6*	ChrX(GRCh37):g.67733173G > A NM_001195214.2:c.62G > A p.(Arg21His) (hemizygous)	Possibly causative, no segregation analysis possible

Variants are in heterozygous state unless stated otherwise

^a^One cell per subject

**Table 3:** Overview of *IKZF2* variants

Family	Variant GRCh37 (hg19) NM_001387220.1	Effect on protein NP_001374149.1	GnomAD minor allele frequency (%)	Variant classification^a^	CADD_ PHRED	SIFT	PolyPhen-2	Mutation Taster
W16-0482	Chr2:g.213914526T > G c.485A > C	p.(His162Pro)	0.0004/0.003 (other/non-Finnish European)	Likely pathogenic (PS2_supp, PS3_supp, PM2, PP3)	24.6	0.00	0.998	Deleterious
W22-1907	Chr2:g.213914502C > T c.509G > A	p.(Cys170Tyr)	–	Likely pathogenic (PS3_supp, PM2, PP1_mod, PP3)	29.4	0.01	0.998	Deleterious
W22-2757	Chr2:g.213914577C > A c.434G > T	p.(Cys145Phe)	–	Unknown significance (PS3_supp, PM2, PP1, PP3)	32.0	0.86	0.998	Deleterious

^a^Variant classification was performed using the American College of Medical Genetics and Genomics Standards and Guidelines (Oza et al. 2018; Richards et al. 2015). CADD, Combined Annotation Dependent Depletion, threshold value ≥ 15 (Kircher et al. 2014); GnomAD, Genome Aggregation Database (version 2.1.1), total population exome frequency / maximum exome frequency (corresponding population); MutationTaster, threshold value ‘deleterious’ (Schwarz et al. 2014); PolyPhen-2, Polymorphism Phenotyping v2, threshold value ≥ 0.450 (Adzhubei et al. 2010); SIFT, Sorting Intolerant From Tolerant, threshold value ≤ 0.05 (Ng and Henikoff 2001); –, not present in database

**Fig. 2:**
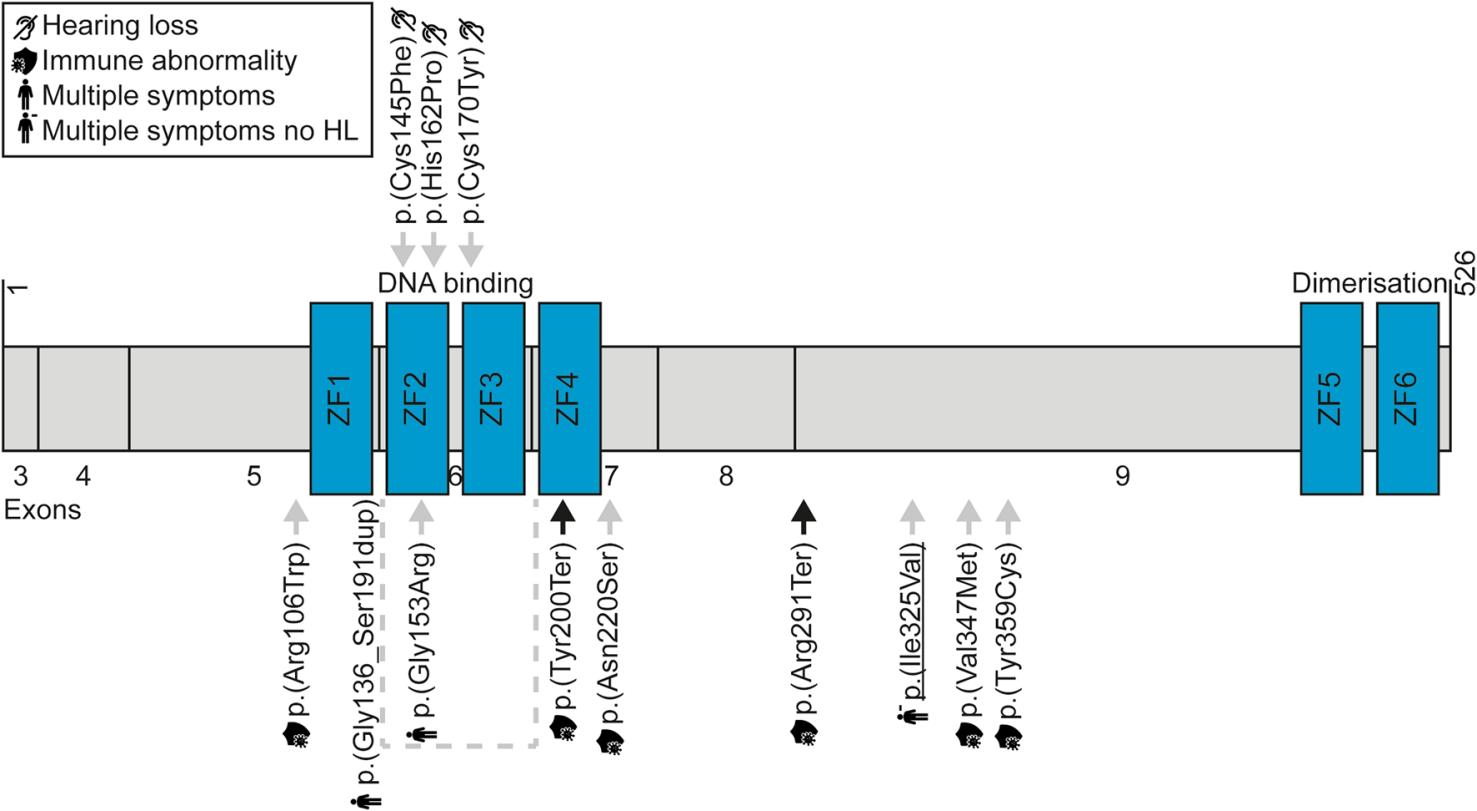
Schematic representation of the Helios protein and its functional domains. Adapted and updated version of a previously published figure by Mohajeri et al. (2023). The variants reported in this study are shown above the protein, while previously reported variants are displayed below the protein. The underlined variant was found to by present homozygously. Grey arrows indicate missense variants, and black arrows indicate stop gain variants. The legend for the symbols used is provided within the figure: ‘Hearing loss’ signifies non-syndromic hearing loss and pertains to the subjects in this study; ‘Immune abnormality’ refers to variants associated with immune deficiency or dysregulation (Hetemäki et al. 2021; Mayr et al. 2022; Shahin et al. 2022); and ‘Multiple symptoms’ indicates syndromic disease, including both hearing loss and immune abnormalities, referencing two patients described by Mohajeri et al. (2023). Exon numbers are according to the reference sequence NM_001387220.1. ZF, zinc-finger motif

#### Candidate genes

After exclusion of variants in twelve genes with a disproportionately high number of variants, 13,033 variants were identified in candidate genes (Supplemental Table 1, lists 2, 3 and 4; Supplemental Table 4). Selection of variants that were potentially bi-allelic revealed 2228 variants. Because the study population consisted of individuals with non-syndromic HL or, at least, without other major health issues, variants in genes associated with other phenotypes in OMIM were excluded. After this step, 1323 variants remained. By using additional in silico prediction tools (CADD_PHRED, SIFT, PolyPhen-2 and MutationTaster) and manually assessing alignment files, 735 and 546 variants were excluded, respectively (again mainly in older files and contiguous nucleotides). Of the remaining 42 variants, four variants were excluded because the subjects decided to withdraw from the study, six because reassessment of clinical information revealed that the cause of HL was unlikely to be genetic and six because the case was solved after inclusion (Supplemental Table 3). Twenty-five variants, of which six in hemizygous state, were selected for follow-up (Table 2; Supplemental Table 5).

**Table 4:** Results of questionnaires and assessment of medical records in affected subjects with *IKZF2* variants

Family	Subject	Sex	Age^a^ (years)	Age of HL onset^b^	Progression^c^	Use of hearing aids	Risk factors for acquired HL	DHI	Other
W16-0482	II:2	M	56	45 years	Yes	Yes	Antibiotic use^d^; noise exposure	0	Tinnitus; positive family anamnesis for rheumatism
	III:2	F	N/a	15 years	MD	MD	MD	MD	
W22-1907	II:3	F	62	12 years	Yes	Yes	Recurrent ear infections	8	Tinnitus; symptoms of vertigo
	II:4	M	53	18 years	Yes	Yes		0	
	III:1	M	39	Childhood	Yes	No	Ear surgery (tubes); recurrent ear infections	2	Tinnitus; medical history of congenital choanal atresia on the left side
	III:2	F	38	Childhood	Yes	No	Antibiotic use; ear surgery (tubes)	14	
	III:3	M	36	Adolescence	Yes	No	MD	MD	
W22-2757	II:2	M	59	32 years	Yes	Yes		0	
	III:1	F	43	3–4 years	Yes	MD	MD	MD	
	III:2	F	38	20–25 years	Yes	Yes	MD	MD	

DHI, dizziness handicap inventory; F, female; HL, hearing loss; M, male; MD, missing data (applicable to retrospective assessment of medical records in case of uncompleted questionnaires); N/a, not applicable; y, years

^a^Age at completing questionnaire (not applicable in case of data retrieval from medical records)

^b^Self-reported age of onset

^c^Self-reported progression

^d^Type of which unknown

Variants in *ANKRD17*, *ELK1*, *ENOX2, HEPH*, *SHPRH, SRPX* and *YIPF6* were considered to be possibly causative to the HL in the relevant subjects (Table 2). For the *ANKRD17*, *ELK1*, *ENOX2*, and *YIPF6* variants, no segregation analysis was possible, e.g. due to the absence of siblings or the unwillingness of relatives to participate. For variants in *HEPH, SHPRH,* and *SRPX*, only limited segregation analysis could be performed, and there were no other subjects with candidate variants in these genes. A hemizygous variant in the X-linked gene *HEPH* was confirmed to be inherited from the unaffected mother of the index case. No other relatives were available for segregation analysis. For the index case with two *SHPRH* variants and mild-to-moderate down-sloping HL in childhood, it was confirmed that these were present *in trans*. However, the absence of siblings precluded further segregation analysis. An unaffected brother of the index case with a hemizygous *SRPX* variant did not have the variant.

So far, there is no additional support for the association with HL for these seven genes with potentially causative variants, neither from mouse variants (IMPC, http://mousephenotype.org (Groza et al. 2023)), MGI (Baldarelli et al. 2024), or literature) nor from direct interactions of the encoded proteins with proteins known to be critical for hearing (STRING database (Szklarczyk et al. 2023)). The remaining candidate variants were excluded because they did not co-segregate with HL, were present *in cis*, or were not validated by PCR and Sanger sequencing (Supplemental Table 5).

#### CNV analysis

Eighty-one monoallelic truncating variants were identified in human deafness genes (Supplemental Table 1, list 1: (non-)syndromic deafness genes) and 581 in candidate deafness genes (Supplemental Table 1, lists 2, 3 and 4) (Supplemental Table 6). These were further addressed if CNV analysis (CoNIFER or ExomeDepth) was performed in clinical exome sequencing. In two samples, a CNV was called in the human deafness gene in which the truncating variant was identified and in twelve samples this was the case for candidate deafness genes. Six of the latter samples were excluded because the gene in which the CNV was called (*MUC17*, *OR2A7*), was known to be highly variable (Lewis et al. 2022). In total, eight samples were selected for confirmation of the CNV by genomic qPCR (Supplemental Table 7). Only a deletion in *MGAM* could be confirmed. This deletion was identified in two index cases who harboured the same truncating *MGAM* variant. Segregation analysis for one of the subjects demonstrated that the truncating variant and the deletion were located on the same allele.

### Variant prioritization in group AD

#### Known human deafness genes

Initially, we identified 91 variants in known human deafness genes in group AD (Supplemental Table 8). Twenty missense variants were excluded based on evaluations by four in silico prediction tools (CADD_PHRED, SIFT, PolyPhen-2 and MutationTaster). Additionally, 18 variants were excluded as they had previously been identified in clinical exome sequencing analyses and could either not be validated (n = 7) or were proven to be non-causal, for example, through segregation analysis (n = 11). Of the remaining 53 variants, 36 were selected for further evaluation based on assessment of alignment files. Subsequently, 29 of these were excluded after phenotype and variant type comparison with the literature (n = 19) or validation of the inheritance pattern (n = 5), using updated MutationTaster predictions (MutationTaster2021 (Steinhaus et al. 2021) (n = 5), or because of insufficient phenotypic information about the subject (Supplemental Table 9). Two variants were excluded for two reasons. Ultimately, seven variants were selected for follow-up. A *TRRAP* variant was considered likely causative but none of the seven variants could be addressed in segregation analysis, shown to co-segregate with HL and/or classified as (likely) pathogenic according to ACMG criteria (Supplemental Table 10 and Supplemental Results).

#### Mouse deafness genes

A total of 787 variants in orthologs of mouse deafness genes were identified, 490 of which were in genes with variants present in at least three samples (Supplemental Table 11). A subgroup of mouse deafness genes was used to select the most promising variants (Supplemental Table 1), resulting in 33 candidate variants. Eighteen of these were excluded upon assessment of alignment files. Fifteen variants in five different genes were selected for follow-up of which only two variants in *IKZF2* remained as strong candidates (Supplemental Table 12; Table 3). These two variants affect highly conserved cysteine or histidine residues in ZF motifs of the encoded protein Helios (Fig. 2).

*IKZF2* has been shown to be crucial for the functional maturation of outer hair cells in mice and, consequently, for hearing (Chessum et al. 2018). This gene encodes Helios, a member of the Ikaros ZF transcription factor family, typically with four ZF motifs in the N-terminal region of the protein and two in the C-terminal region (Fig. 2, Mohajeri et al. 2023).

### Further assessment of candidate gene *IKZF2*

#### Evaluation of *IKZF2* variants in additional exomes

To strengthen the findings for *IKZF2*, variants in this gene were assessed in unresolved cases with presumed HHL who underwent clinical exome sequencing after the initiation of the present study. This revealed two additional rare missense variants. The first variant was derived from the affected father (family W22-2757) and, interestingly, also affects a zinc-binding residue in a ZF motif of Helios (Fig. 2, Table 3). Consequently, this variant was included for further assessment. The second variant, Chr2(GRCh37):g.213872179G > A NM_001387220.1:c.1486C > T p.(Arg496Trp), did not co-segregate with HL in the family.

#### The identified *IKZF2* variants

**Fig. 3:**
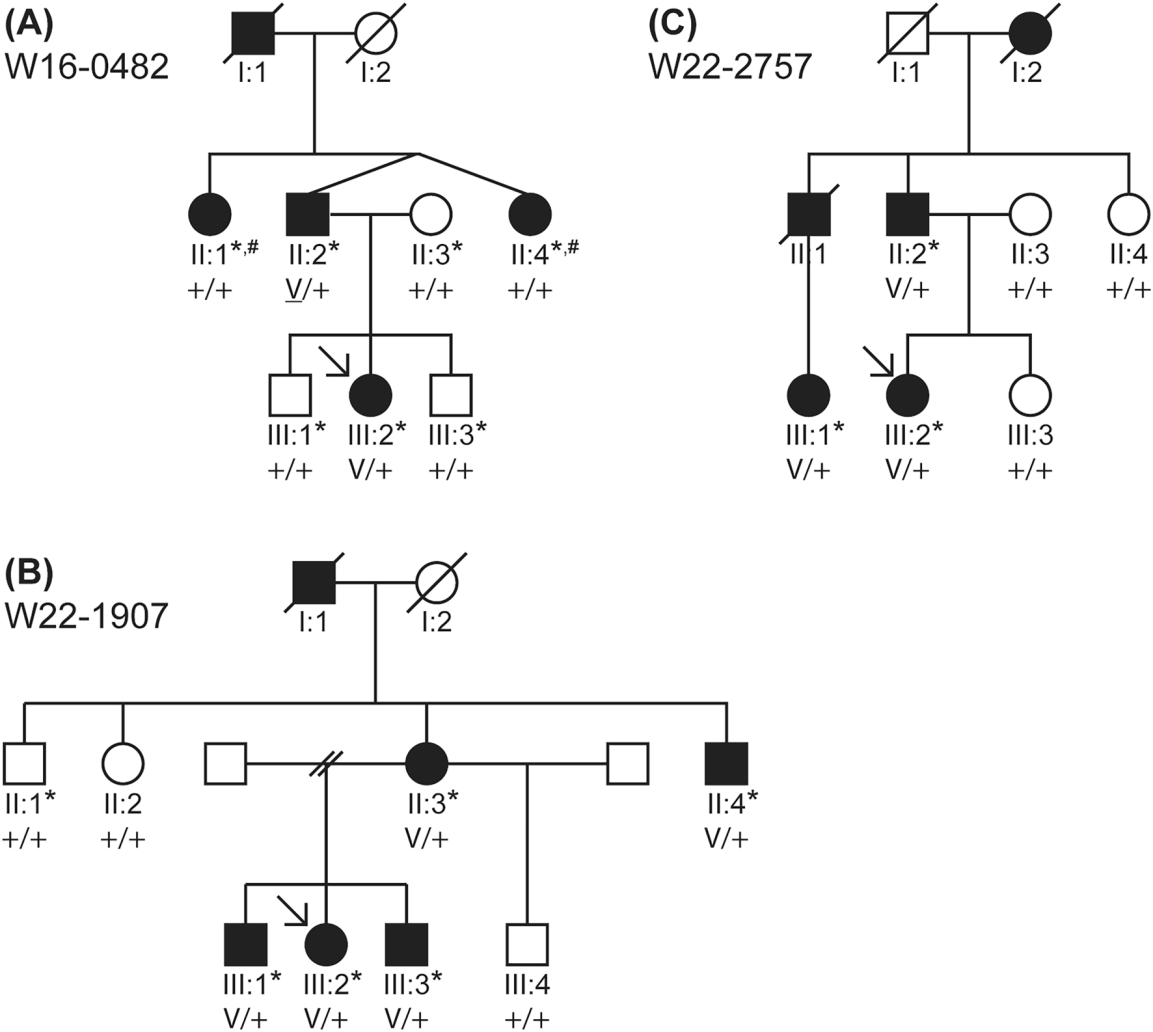
Pedigrees of families W16-0482, W22-1907 and W22-2757 and co-segregation of the identified *IKZF2* variants with HL. The clinical status of the individuals is indicated by the filling of the symbols: black indicates that the subject has HL, and white indicates that the subject has no HL. The clinical status is based on audiograms in all subjects marked with an asterisk. **A** Family W16-0482. V: c.485A>C p.(His162Pro). Subjects II:1 and II:4 (marked with a hashtag) did have HL, but a significantly milder phenotype than other affected family members. The variant (V) was found to be de novo in II:2 (Supplemental Fig. 2). **B** Family W22-1907. V: c.509G>A p.(Cys170Tyr). **C** Family W22-2757. V: c.434G>T p.(Cys145Phe). + , wildtype; square, male; circle, female; slash through symbol, deceased; arrow, proband

The three variants in *IKZF2* to be further addressed were Chr2(GRCh37):g.213914526 T > G NM_001387220.1:c.485A>C NP_001374149.1:p.(His162Pro) (ACMG classification ‘likely pathogenic’: PS2_supp, PS3_supp, PM2, PP3) in family W16-0482, Chr2(GRCh37):g.213914502C>T NM_001387220.1:c.509G>A NP_001374149.1:p.(Cys170Tyr) (ACMG classification ‘likely pathogenic’: PS3_supp, PM2, PP1_mod, PP3) in family W22-1907 and Chr2(GRCh37):g.213914577C>A NM_001387220.1:c.434G>T NP_001374149.1:p.(Cys145Phe) (ACMG classification ‘unknown significance’: PS3_supp, PM2, PP1, PP3) in family W22-2757 (Fig. 3, Table 3; Supplemental Fig. 1). All three variants were predicted to be deleterious by either three or all four of the used in silico tools and were either absent in gnomAD (version 2.1.1) or present only at a frequency < 0.01% (Table 3).

None of the variants were predicted to impact splicing according to SpliceAI. In line with the ACMG Standards and Guidelines and its specifications for hearing loss (Oza et al. 2018; Richards et al. 2015), intended for diagnostic use, all three variants were classified as variants of unknown significance. Interestingly, each of these three missense variants impacts one of the four critical amino acid residues for Zn-binding, i.e., cysteine or histidine. These are arranged in a highly conserved Cys2-His2 (C2H2) ZF motif, of which the ZF protein Helios contains six, interspersed by disordered regions. Four N-terminal domains are DNA-binding, and two C-terminal domains are protein interaction domains (Powell et al. 2019). Specifically, the p.(Cys145Phe) and p.(His162Pro) variants affect the second N-terminal ZF motif and the p.(Cys170Tyr) variant affects the third N-terminal ZF motif (Fig. 2).

#### Segregation analyses of *IKZF2* variants

To confirm the association of *IKZF2* variants with the HL in the families, relatives were included in the study.

**Fig. 4:**
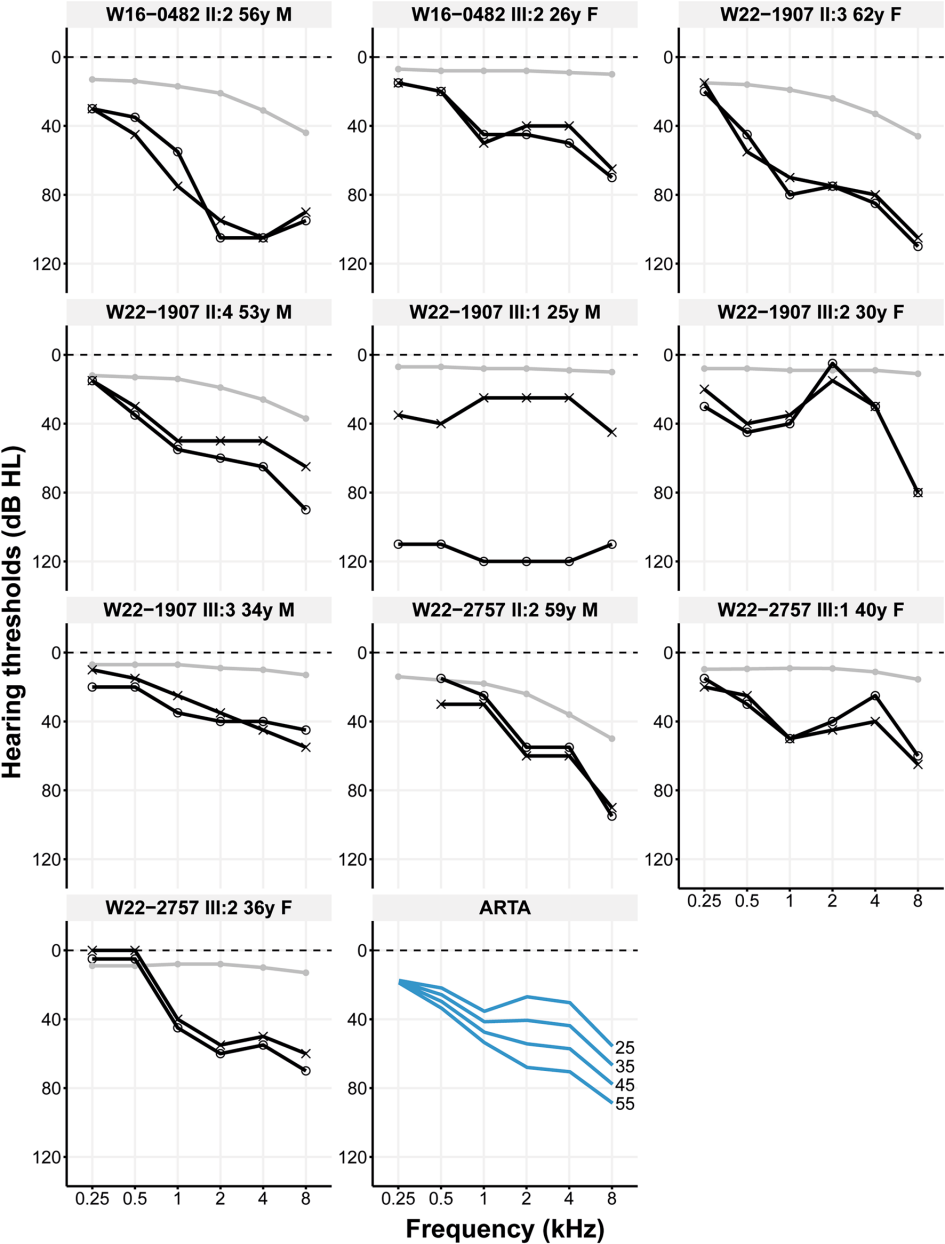
Audiological features of subjects with *IKZF2* variants. The first ten panels show the pure tone air conduction thresholds in dB HL of 0.25–8 kHz of subjects identified with an *IKZF2* variant (c.485A>C p.(His162Pro) in family W16-0482; c.509G>A p.(Cys170Tyr) in family W22-1907; c.434G>T p.(Cys145Phe) in family W22-2757) from whom audiometric data was available. Black lines with circles represent the right ear, black lines with crosses represent the left ear, grey lines and dots represent the age- and gender-specific 95th percentile. The last panel shows the age-related typical audiograms (ARTA), derived from cross-sectional linear regression analysis of the most recent audiograms of individuals identified with *IKZF2* variants. Each blue line represents a ten-year age span. dB HL, decibel hearing level; F, female; kHz, kilo Hertz; M, male; y, years

The index case (III:2) in family W16-0482 (Fig. 3A) inherited the c.485A>C p.(His162Pro) variant from her affected father (II:2), unlike her two unaffected brothers. The variant was not identified in two paternal aunts (subjects II:1 and II:4) of the index case, both of whom exhibited bilateral SNHL. However, with a better ear PTA_0.5–4 kHz_ 25 dB HL (II:1) and 39 dB HL (II:4), respectively, their HL was markedly milder than that of their brother (II:2, 75 dB HL) and his daughter (III:2; 38 dB HL at 26 years of age) who both had the variant. (Fig. 4, Supplemental Fig. 2, Supplemental Table 13). Therefore, we hypothesized that SNHL in this family was genetically heterogeneous. VNTR marker analysis revealed that subjects II:2 and II:4 shared the same genotype for markers flanking *IKZF2* despite the discordance in the nucleotide at position c.485 (Supplemental Fig. 3). The latter was confirmed by sequencing two different amplicons of the relevant exon to rule out that only the WT allele was amplified for subject II:4. These results suggest that the *IKZF2* variant occurred de novo in subject II:2. To explore other potential causes of HL in all affected family members, exome sequencing was performed for subjects II:1, II:2, III:1 and III:2 using the same exome enrichment kit and sequencing platform. Variants in known deafness genes (list 1) as well as in candidate deafness genes (lists 2, 3 and, 4) present in subjects II:1, II:2 and III:2, but absent in subject III:1, were assessed but did not yield an alternative explanation for the HL. The reported age of onset of subject II:2, was 45 years, and that of subject III:2 15 years. Only subject II:2 completed the questionnaire, he described the HL as progressive, and the DHI score was 0, indicating no-to-mild vestibular dysfunction.

For family W22-1907, seven relatives of the proband (III:2) participated in this study (Fig. 3B). All four relatives (II:3, II:4, III:1, III:3) with bilateral SNHL, and none of the three subjects with normal hearing (II:1 (Supplemental Fig. 2), II:2 and III:4), were found to be heterozygous for the c.509G>A p.(Cys170Tyr) variant. Better ear PTA_0.5-4 kHz_ ranged from 29 to 30 dB HL for subjects in generation III (aged 25–34), while those for subjects in generation II (aged 53 and 62) were 45 and 70 dB HL, respectively (Fig. 4, Supplemental Table 13). Only subject III:1 had a considerably asymmetrical, progressive HL with hearing thresholds of 29 dB HL in the left ear and a functionally deaf ear on the right (118 dB HL). Since the HL in the right ear manifested much earlier than that in the left ear, and given the greater severity of the HL in the right ear compared to both the left ear and other subjects, the HL in subject III:1’s right ear was considered unrelated to the *IKZF2* variant. Medical examinations failed to explain the severe HL in this subject’s right ear. Specifically, there was no history of meningitis or head trauma, and otoscopy and CT imaging did not reveal any (anatomic) abnormalities, except for a choanal atresia on the left side, which was deemed to be unrelated. No MRI had been performed. The reported age of onset was 12 (II:3), 18 (II:4), in childhood (III:1, III:2), and in adolescence (III:3) and the HL was progressive (Table 4). The available DHI scores were 0 (II:4), 2 (III:1), 8 (II:3) and 14 (III:2), all indicating no-to-mild vestibular dysfunction.

For family W22-2757, two affected relatives of the proband (III:2) were included in this study, and three with normal hearing (Fig. 3C). Genotyping confirmed co-segregation of the c.434G>T p.(Cys145Phe) variant with HL in the family. The proband inherited the variant from her father (II:2), and both subjects had bilateral, symmetrical SNHL (Fig. 4). For the proband, the better ear PTA_0.5-4 kHz_ was 36 dB HL at the age of 36, while that of her father (II:2, age 59) was 38 dB HL (Supplemental Table 10). The variant was also identified in her affected cousin, with a better ear PTA_0.5-4 kHz_ of 36 dB HL at the age of 40. The reported ages of onset were 3–4 years (III:1), 20–25 years (III:2) and 32 years (II:2). All subjects reported progressive HL. Subject II:2 completed the DHI questionnaire and scored 0, indicating no-to-mild vestibular dysfunction.

The mean difference in PTA_0.5-4 kHz_ between the better and worse ears for all affected subjects was 4 dB HL, except for subject III:1 from family W22-1907 with asymmetrical HL. None of the subjects with HL and variants in *IKZF2* nor their affected relatives reported symptoms of other medical conditions.

#### Protein structural analysis

**Fig. 5:**
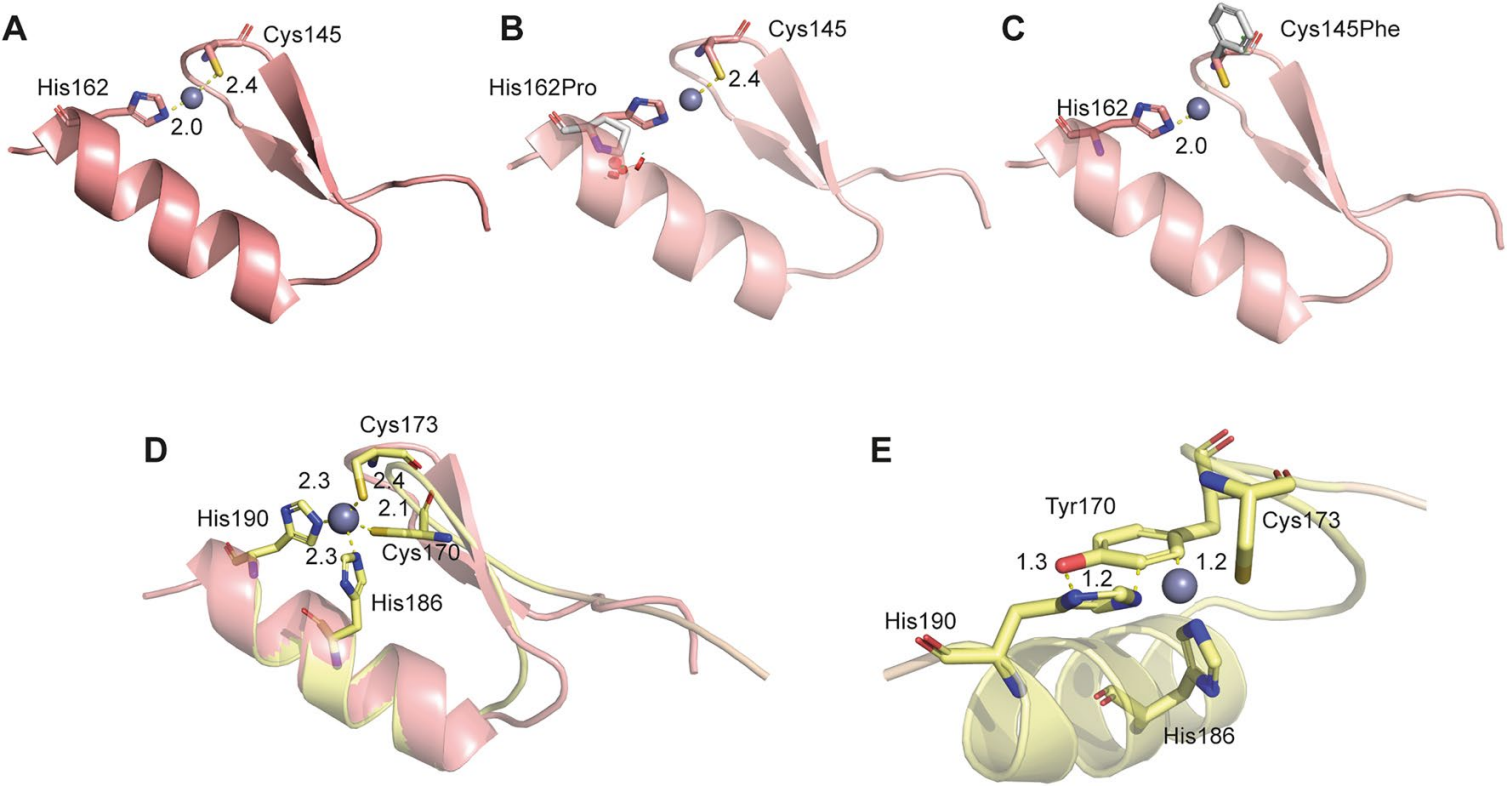
Structural modeling of *IKZF2* variants. **A** Crystal structure of the second N-terminal C2H2 domain (PDB: 7LPS) is illustrated, with the variant positions of interest His162 and Cys145 highlighted in sticks at the Zn^+2^ coordination site. Atomic distances shown are in angstroms (Å). Missense variants c.485A>C p.(His162Pro) and c.434G>T p.(Cys145Phe) represented as grey sticks are depicted in (**B**, **C**), respectively. **D** The third variant position of interest, Cys170, is located at the third N-terminal C2H2 domains Zn^+2^ coordination site, as depicted. The figure shows superposition of the modelled structue of the third C2H2 domain (yellow, with side chains optimized) onto the second C2H2 domain (light red) cocrystallized with Zn^+2^. **E** The missense variant c.509G>A p.(Cys170Tyr) is predicted to cause steric clashes in its neighbourhood, indicated by the non-bonded distances that are less than the sum of their van der Waals radii (< 1.8 Å)

Based on structural data, the two missense variants c.485A>C p.(His162Pro) and c.434G>T p.(Cys145Phe) were found to be located at the zinc ion (Zn^2+^) coordination site of the second N-terminal Zn finger (Fig. 5A). The primary role of Zn^+2^ ions is to impart structural stability. Structural modelling of the two variants suggests considerable protein destabilization due to disruption of Zn^2+^ coordination: ∆∆G_mut-wt_ His162Pro = 6.88 kcal/mol, ∆∆G_mut-wt_ Cys145Phe = 6.62 kcal/mol. Both variants are anticipated to cause loss of helical structural integrity as proline (c.485A>C p.(His162Pro)) can neither form a helix nor interact with Zn^2+^, and phenylalanine (c.434G>T p.(Cys145Phe)) loses Zn^2+^ coordination due to the aromatic and hydrophobic side chains of Phe145 (Fig. 5B, C, variants in grey sticks). Both missense variants are thus predicted to disrupt protein folding, thereby affecting protein function. Furthermore, we assessed variant c.509G>A p.(Cys170Tyr) present in the third N-terminal C2H2 domain in a similar manner. Structural superposition of the modelled structure of the third C2H2 domain onto the second C2H2 domain, indicated the presence of the variant at the Zn^2+^ coordination site marked by Cys170, Cys173, His186, and His190 (Fig. 5D). The substitution of Cys170 to a bulky and aromatic tyrosine is expected to disrupt the structural integrity of Helios as well, through steric clashes (Fig. 5E), resulting in the loss of ability to bind Zn^2+^, ∆G_mut_ − ∆G_wt_ Cys170Tyr = 10.75 kcal/mol. The structural analyses of the three missense variants are thus suggestive of deleterious functional outcomes through misfolded C2H2 domains.

#### Functional evaluation of *IKZF2* variants

**Fig. 6:**
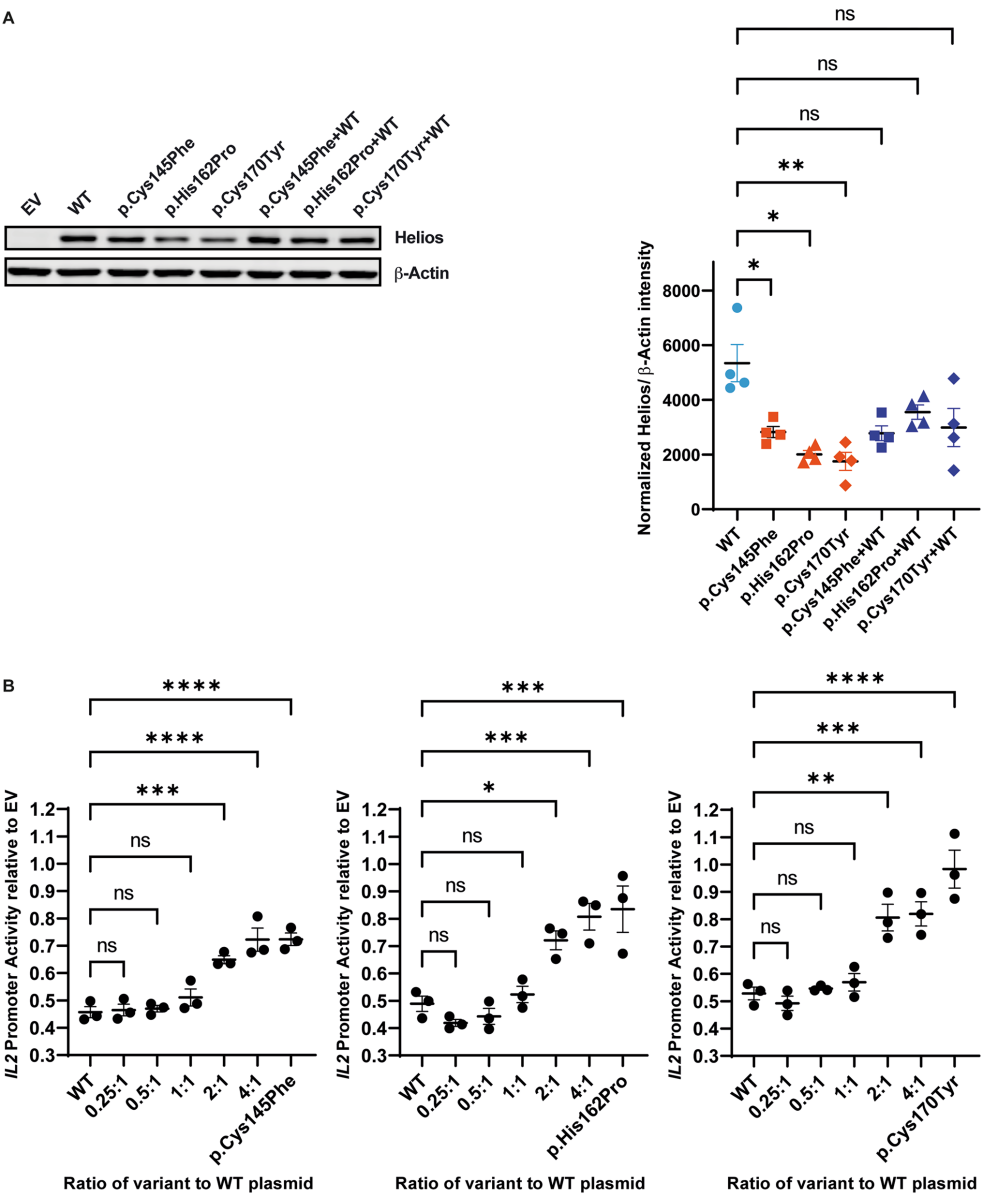
Functional evaluation of the identified *IKZF2* variants. Variant c.485A>C p.(His162Pro) in family W16-0482, variant c.509G>A p.(Cys170Tyr) in family W22-1907 and variant c.434G>T p.(Cys145Phe) in family W22-2757. **A** Assessment of variant protein expression in HEK293 cells. Immunoblot analysis of cells transfected with plasmids coding for 3xFLAG-tagged WT Helios or Helios with the amino acid substitutions alone, or a combination of WT and variant plasmids. There is a statistically significant decrease in Helios protein levels when the variants are expressed alone but no significant decrease when the Helios variants are co-expressed with WT Helios (right panel). A parametric one-way ANOVA was used for statistical analysis: *p < 0.05; **p < 0.01. **B** Luciferase reporter assays to assess the variants’ ability to repress *IL2* promotor activity, demonstrating statistically significant decrease in repression of the *IL2* promotor by the variant Helios proteins when expressed alone. Upon co-expression of WT and variant Helios in increasing ratios of transfected variant plasmids, a statistically significant decrease of *IL2* promoter repression is only observed for ratios of 2:1 and higher. A parametric one-way ANOVA was used for statistical analysis: *p < 0.05; **p < 0.01; ***p < 0.001; ****p < 0.0001. EV, empty vector control; *IL2*, interleukin-2; ns, not statistically significant; WT, wildtype

To assess the effect of the amino acid substitutions on Helios expression, HEK293 cells, which lack basal expression of Helios, were transfected with p.CMV6-XL4 plasmids encoding FLAG-tagged WT or either of the three variant Helios proteins. Additionally, considering the heterozygous nature of the variants in the patients, we aimed to mimic the in vivo conditions more closely and assess whether the WT and variant forms of Helios have synergistic, antagonistic, or independent effects on each other's expression in the cellular environment. To achieve this, cells were co-transfected with equal proportions of the WT and each variant *IKZF2* plasmid. An EV was used as a negative control. In Western blot analysis, all three Helios variants, when expressed individually, demonstrated significantly lower expression levels than WT Helios (Fig. 6A). This indicates an impact of the amino acid substitutions on protein synthesis and/or stability. In contrast, cells co-transfected with WT and each respective variant plasmid exhibited a reduction in expression relative to WT alone; however, this reduction was not statistically significant (Fig. 6A).

To study the functional impact of the variants, we employed a well-established luciferase reporter assay that has previously been used to demonstrate a dominant-negative effect of variants in *IKZF2* (Mohajeri et al. 2023). In this assay, HEK293 cells were co-transfected with a firefly luciferase reporter plasmid harbouring the − 580 to + 57 region of the human *IL2* promoter, which includes the Helios DNA binding consensus sequence, along with either EV, WT, or variant *IKZF2* plasmids. Results indicated that each variant significantly reduced the ability to repress transcription from the *IL2* promoter when compared to WT. This reduction was statistically significant for all three variants. In the co-transfection assays with ratios of 0.25:1, 0.5:1 and 1:1 variant to WT plasmids, no significant difference was observed in *IL2* repression when compared to WT alone (Fig. 6B). However, at ratios of 2:1 and higher, there was a notable decrease in suppressive activity, as evidenced by an S-shaped curve in the graphical representation (Fig. 6B), suggesting a compromised ability to suppress *IL2* expression at these higher variant-to-WT ratios.

## Discussion

In this study, exome sequencing data were assessed to identify the causes of HL in a cohort of 461 index cases with presumably non-syndromic hereditary SNHL and an onset before the age of 50. These index cases remained unsolved after medical genetic testing by clinical exome sequencing combined with targeted gene panel analysis for HL between 2013 and 2020. The main goal of the study was to identify novel genes for HL by designing a strategy to prioritize DNA-variants in candidate genes for further analysis. However, the first step in the analysis was to address variants in genes of the most recent gene panel (354 genes, list 1) because the number of genes associated with HL significantly increased since 2013 (Hearing Impairment Gene Panel DGD181213, containing 102 genes).

The designed strategy and follow-up of variants resulted in a (likely or potential) genetic diagnosis for five families. These represent 1.1% of the index cases in our total cohort, 3.2% in the AD group and 0.3% in the AR group. A single novel gene for non-syndromic HL was identified, *IKZF2*, with missense variants co-segregating with autosomal dominantly inherited HL in three families. The outcome of the analysis strategy did not meet our expectations, especially for the AR group. There are several potential explanations for this limited number of diagnoses. Obviously, causative variants affecting intronic and/or regulatory regions of genes or regions with insufficient sequencing coverage were lacking from the data. The latter is expected to be more common in the oldest exome sequencing data. Furthermore, CNV detection in exome sequencing data is not optimal, which is even more the case for other types of structural variants (SVs) (de Bruijn et al. 2021; Porubsky and Eichler 2024). Targeted analyses using genome sequencing or other techniques, mainly applied for monoallelic cases, have already demonstrated the occurrence of pathogenic SVs, e.g., in *USH2A* and *PCDH15* (Reurink et al. 2023; Vaché et al. 2020). For *STRC* and *OTOA*, with neighbouring pseudogenes, SVs are the most prominent pathogenic variants (Domínguez-Ruiz et al. 2023; Laurent et al. 2021). Ongoing and future short-read and, specifically, long-read genome sequencing analyses will likely unveil the overall importance of SVs in known and to-be-identified genes for HL. However, the added overall diagnostic yield of short-read genome sequencing was reported to be limited (~ 8%) when compared to that of exome sequencing (Wojcik et al. 2024). Currently, high fidelity long-read genome sequencing can reveal the most comprehensive variant dataset when using a single technology (Kucuk et al. 2023) but to be implemented as a first-tier test, its costs need to decrease substantially.

A further reason for missing the causative genetic defects is that the assumed inheritance pattern of HL was incorrect. Isolated cases in the AR group, for example, might be explained by monoallelic de novo variants. This has been shown previously for HL (Smits et al. 2019; Wesdorp et al. 2018) and is substantiated by Klimara et al., who described that de novo variants were causative in at least ∼1% of probands for whom a genetic diagnosis was established (Klimara et al. 2022). Also, the genetic heterogeneity of HL in families and/or the presence of phenocopies may have masked the true inheritance pattern of the condition.

Finally, the low percentage of diagnoses might well be due to deselection of the relevant genes and variants in our approach. This includes the decision to exclude genes linked to disease in OMIM in the AR group, despite the possibility that variants in the same genes can lead to different disorders, as shown here for *IKZF2*. The main reason for applying the stringent strategy for variant prioritization and thereby reduction of candidate variants is the effort needed for follow-up in families to confirm co-segregation of variants with HL. The recruitment of family members for both genotyping and phenotyping after the clinical diagnostic phase turned out to be time-consuming and often with limited or no success. Therefore, the inclusion of parents of infants and young adults already in medical genetic testing and/or of additional family members, especially in case of dominantly inherited HL, is highly recommended. This will not only facilitate variant interpretation in medical genetic testing but also aid in the identification of novel genes for HL.

Besides inclusion of family members in medical genetic testing, an improved strategy for the selection of candidate variants could increase the probability of co-segregation with HL and enable the assessment of all genes. Greene et al. (2023) designed such a strategy and applied the Bayesian genetic association method BeviMed to rare variants in coding genes to identify genetic aetiologies of rare diseases. Genomes of 77,539 individuals from the 100,000 Genomes Project were used, including family members as well as controls. For HL, this strategy was applied to about 700 probands with hearing/ear disorders, the vast majority with non-syndromic HL. Two strong novel associations with HL were identified, *GPR156* and *FMN1*, in addition to fifteen known gene associations. Increasing the number of cases by combining different cohorts of presumed HHL and controls could increase the power to identify novel genes for HL.

In the AD group, the strategy applied in the present study associated *IKZF2* with non-syndromic SNHL. The *IKZF2*-encoded Helios is a member of the Ikaros family of ZF transcription factors together with the namesake Ikaros (*IKZF1*) and Aiolos (*IKZF3*), Eos (*IKZF4*) and Pegasus (*IKZF5*). Their function has been studied in neurodevelopment but most extensively in hematopoietic cell lineages (Alsiö et al. 2013; Giralt et al. 2020; Martín-Ibáñez et al. 2017; Read et al. 2021 and references therein). This is partly due to their association with inborn errors of the immune system, thrombocytopenia, and/or predisposition to leukaemia (*IKZF1* and *IKZF3*) and to somatic defects that are frequently occurring in haematological malignancies (*IKZF1* and *IKZF2*) (Kuehn et al. 2016; Lentaigne et al. 2019; Park et al. 2019; Shahin et al. 2022; Yamashita et al. 2021).

Monoallelic germline defects of *IKZF2* have been found to underlie immunodeficiencies and autoimmunity (with incomplete penetrance), and a biallelic *IKZF2* missense variant was found to cause syndromic immunodeficiency with osteopenia and hypothyroidism (Hetemäki et al. 2021; Shahin et al. 2021, 2022). During the present study of *IKZF2*, HL was also associated with *IKZF2* since monoallelic variants affecting ZFs 3 and/or 2, were described in ICHAD syndrome (**I**mmunodysregulation, **C**raniofacial anomalies, **H**earing impairment, **A**thelia and **D**evelopmental delay) (Mohajeri et al 2023). These phenotypes suggest a complex genotype–phenotype correlation for *IKZF2*. A dominant-negative effect has been proposed for the two variants associated with ICHAD syndrome (Mohajeri et al 2023). In contrast, luciferase assays for Helios with the amino acid substitutions causing non-syndromic HL did not indicate such a dominant-negative effect. For the p.(His162Pro) variant, this is consistent with previous findings for the p.(His167Arg) variant of Ikaros (*IKZF1*) affecting the homologous residue of Helios His162 (Kuehn et al. 2016). However, a loss-of-function effect has been indicated for monoallelic truncating *IKZF2* variants underlying isolated disorders of the immune system, suggesting haploinsufficiency as the underlying disease mechanism. The encoded proteins are either unstable (Hetemäki et al. 2021) or cannot homo- or hetero-dimerize with other Ikaros family members that are co-expressed in hematopoietic cell lineages (Shahin et al. 2022). Missense variants associated with such disorders, and that affect the region between the N-terminal DNA-binding and the C-terminal dimerization ZFs, are also suggested to have a loss-of-function effect since they disrupt or decrease interactions with members of the NuRD complex (Shahin et al. 2021, 2022). This complex is recruited by Helios to specific chromosomal targets and functions in repression or activation of transcription (Sridharan & Smale 2007).

Remarkably, HL has only been diagnosed in cases with variants directly affecting ZFs 2 and 3 (Mohajeri et al. 2023). Also, none of the cases (aged 2–63 years) with an immune dysfunction disorder due to monoallelic *IKZF2* variants with a loss-of-function effect was indicated to have impaired hearing. Therefore, non-syndromic HL caused by the missense variants identified in the present study is unlikely to be due to an (incomplete) loss of function effect, although reduced protein synthesis or stability upon expression in HEK293 cells suggested such an effect. This is in line with the absence of symptoms of immunodeficiency or -dysregulation in the cases with non-syndromic HL. Therefore, we hypothesize that the underlying missense variants have an inner ear-specific effect, which might be of dominant-negative nature but less severe than that of variants associated with congenital HL as part of ICHAD syndrome. This hypothesis is supported by the recessive inheritance of HL in the *cello* mouse with the *Ikzf2* p.(His517Glu) variant. This variant has a loss of function effect as it affects a Zn^2+^ coordinating residue of the 2nd C-terminal ZF with a deleterious effect on protein dimerization (Chessum et al. 2018).

It is tempting to speculate that Helios co-functions with Pegasus (*IKZF5*) in the inner ear. In mice, *IKZF5* is expressed in hair cells in early postnatal development (at low levels), as well as in the adult stage (Cai et al. 2015; Elkon et al. 2015; Liu et al. 2018; Orvis et al. 2021) and is critical for hearing (Bowl et al. 2017). Future studies are needed to address whether the (inner ear) function of dimers of variant Helios and WT Helios or Pegasus is indeed dominant-negatively affected by the missense variants identified in this study. Also, introducing the variants in mice can shed further light on the molecular function of *IKZF2* in the inner ear.

In conclusion, a candidate gene-based strategy was designed to prioritize DNA-variants derived from exome sequencing to identify ‘novel’ genes associated with non-syndromic HL. Applied to a cohort of approximately 460 index cases, this strategy associated specific missense variants in *IKZF2* with dominantly inherited progressive non-syndromic HL. Identification of more affected patients is required to fully define the emerging complex phenotype between *IKZF2* variants and the range of clinical phenotypes. The limited number of genetic diagnoses obtained using the designed strategy indicates that future studies addressing the missing aetiologies of HL would benefit from optimizing variant selection, utilizing larger patient cohorts, and inclusion of family members early in the process. Additionally, the ongoing implementation of genome sequencing in medical genetic testing will not only increase the percentage of cases receiving a diagnosis but will also further support the identification of ‘novel’ genes for HL.

## Supplementary Information

Below is the link to the electronic supplementary material.

**Figure 1.** *IKZF2* sequence analyses for probands of three families. **A** Reference sequence (NM_001387220.1) of *IKZF2* genomic position 213,914,486 (left) to 213,914,604 (right). **B** Sequence analysis of subject III:2 of family W16-0482. **C** Sequence analysis of subject III:2 of family W22-1907. **D** Sequence analysis of subject III:2 of family W22-2757 (JPG 789 KB)

**Figure 2.** Audiological features of **A** subjects with likely causative variants in known human deafness genes and **B** in family members of *IKZF2* families in whom no *IKZF2* variants were identified. Pure tone air conduction thresholds in dB HL of 0.25 to 8 kHz for all subjects who were not identified with an *IKZF2* variant and from whom audiometric data was available. Black lines with circles represent the right ear, black lines with crosses represent the left ear, grey lines and dots represent the age- and gender-specific 95th percentile. dB HL, decibel hearing level; F, female; kHz, kilo Hertz; M, male; y, years (JPG 783 KB)

**Figure 3.** Variable number of tandem repeat marker analysis in family W16-0482. Four VNTR markers were used (D2S2322, D2S1380, D2S334, D2S143). D2S1380 (marked with an asterisk) is located in *IKZF2*. Potential combinations of alleles in generation I, derived from generation II, are shown. See Fig. 3A for a more detailed presentation of the W16-0482 pedigree (JPG 693 KB)

Supplementary file4 (DOCX 73 KB)

Supplementary file5 (DOCX 13 KB)

Supplementary file6 (DOCX 16 KB)

Supplementary file7 (DOCX 13 KB)

Supplementary file8 (DOCX 15 KB)

Supplementary file9 (DOCX 13 KB)

Supplementary file10 (DOCX 15 KB)

Supplementary file11 (DOCX 13 KB)

Supplementary file12 (DOCX 16 KB)

Supplementary file13 (DOCX 14 KB)

Supplementary file14 (DOCX 13 KB)

Supplementary file15 (DOCX 15 KB)

Supplementary file16 (DOCX 19 KB)

Supplementary file17 (DOCX 13 KB)

## Data Availability

The datasets generated during and/or analysed during the current study are available from the corresponding author on reasonable request.
