# Supplementary figures and images for: Exome variant prioritization in a large cohort of hearing-impaired individuals indicates IKZF2 to be associated with non-syndromic hearing loss and guides future research of unsolved cases
Source: Hum Genet. 2024 Oct 16;143(11):1379–99. doi: 10.1007/s00439-024-02706-w (PMC11522133; doi:10.1007/s00439-024-02706-w)

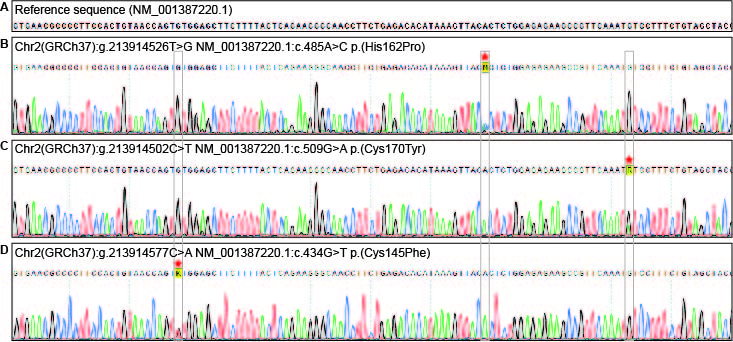

Supplement: Supplementary file 1 — Figure 1. IKZF2 sequence analyses for probands of three families. A Reference sequence (NM_001387220.1) of IKZF2 genomic position 213,914,486 (left) to 213,914,604 (right). B Sequence analysis of subject III:2 of family W16-0482. C Sequence analysis of subject III:2 of family W22-1907. D Sequence analysis of subject III:2 of family W22-2757 (JPG 789 KB) [file 439_2024_2706_MOESM1_ESM.jpg]

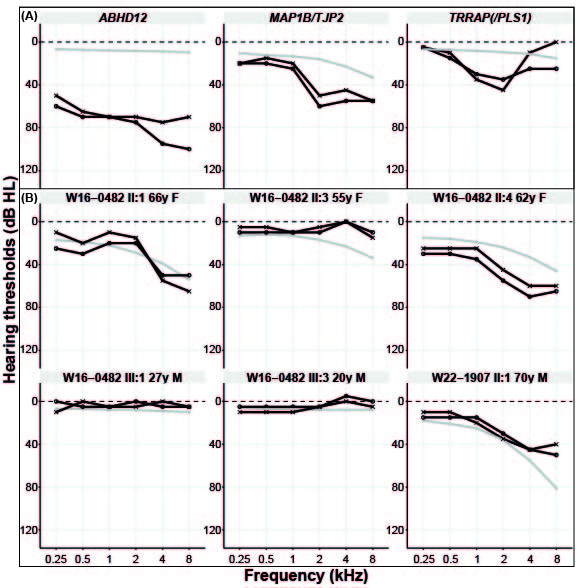

Supplement: Supplementary file 2 — Figure 2. Audiological features of A subjects with likely causative variants in known human deafness genes and B in family members of IKZF2 families in whom no IKZF2 variants were identified. Pure tone air conduction thresholds in dB HL of 0.25 to 8 kHz for all subjects who were not identified with an IKZF2 variant and from whom audiometric data was available. Black lines with circles represent the right ear, black lines with crosses represent the left ear, grey lines and dots represent the age- and gender-specific 95th percentile. dB HL, decibel hearing level; F, female; kHz, kilo Hertz; M, male; y, years (JPG 783 KB) [file 439_2024_2706_MOESM2_ESM.jpg]

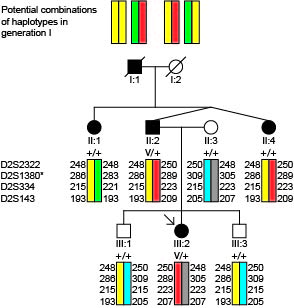

Supplement: Supplementary file 3 — Figure 3. Variable number of tandem repeat marker analysis in family W16-0482. Four VNTR markers were used (D2S2322, D2S1380, D2S334, D2S143). D2S1380 (marked with an asterisk) is located in IKZF2. Potential combinations of alleles in generation I, derived from generation II, are shown. See Fig. 3A for a more detailed presentation of the W16-0482 pedigree (JPG 693 KB) [file 439_2024_2706_MOESM3_ESM.jpg]
