# Supplementary material for: Exome variant prioritization in a large cohort of hearing-impaired individuals indicates IKZF2 to be associated with non-syndromic hearing loss and guides future research of unsolved cases
Source: Hum Genet. 2024 Oct 16;143(11):1379–99. doi: 10.1007/s00439-024-02706-w (PMC11522133; doi:10.1007/s00439-024-02706-w)
Supplement: Supplementary file 4 — Supplementary file4 (DOCX 73 KB) [file 439_2024_2706_MOESM4_ESM.docx]

**Supplemental Table 1**. Gene lists that were used for analyses.

| **Human deafness genes (list 1)** | | | **Orthologs of mouse deafness genes (list 2)** | **Preferential inner ear expression genes (list 3)** | | | **Other candidate genes (list 4)** |
| --- | --- | --- | --- | --- | --- | --- | --- |
| **Non-syndromic deafness genes** | **Syndromic deafness genes** | **Additional OMIM deafness genes** |  | **Schrauwen *et al.* candidate genes** | **Morton substracted candidate genes** | **cDNA bank new deafness genes** |  |
| *ABCC1*  *ACTG1**  *ADCY1*  *AIFM1*  *ATOH1*  *ATP2B2*  *BDP1*  *CABP2*  *CCDC50*  *CD164*  *CDC14A*  *CDH23**  *CEACAM16*  *CIB2**  *CLDN14*  *CLDN9*  *CLIC5*  *CLRN2*  *COCH*  *COL11A1**  *COL11A2**  *COL4A6*  *CRYM*  *DCDC2*  *DIABLO*  *DIAPH1*  *DMXL2**  *DSPP*  *ELMOD3*  *EPS8*  *EPS8L2*  *ESPN**  *ESRP1*  *ESRRB*  *EYA4**  *FOXF2*  *GAB1*  *GAS2*  *GIPC3*  *GJB2*  *GJB3*  *GJB6*  *GPSM2**  *GRAP*  *GREB1L*  *GRHL2**  *GRXCR1*  *GRXCR2*  *GSDME*  *HGF*  *HOMER2*  *IFNLR1*  *ILDR1*  *KARS**  *KCNQ4*  *KITLG**  *LHFPL5*  *LMX1A*  *LOXHD1*  *LRP5*  *LRTOMT*  *MAP1B*  *MARVELD2*  *MCM2*  *MET*  *MIR96*  *MPZL2*  *MSRB3*  *MYH14**  *MYH9**  *MYO15A*  *MYO3A*  *MYO6**  *MYO7A**  *NARS2**  *NCOA3*  *NLRP3**  *OSBPL2*  *OTOA*  *OTOF*  *OTOG*  *OTOGL*  *P2RX2*  *PCDH15**  *PDE1C*  *PDZD7*  *PI4KB*  *PJVK*  *PLS1*  *PNPT1**  *POU3F4*  *POU4F3*  *PPIP5K2*  *PRPS1**  *PSIP1*  *PTPRQ*  *RDX*  *REST*  *RIPOR2*  *ROR1*  *S1PR2*  *SCD5*  *SERPINB6*  *SIX1**  *SLC12A2**  *SLC17A8*  *SLC22A4*  *SLC26A4**  *SLC26A5*  *SLC44A4*  *SMPX*  *SPNS2*  *STRC*  *SYNE4*  *TBC1D24**  *TECTA*  *THOC1*  *TJP2*  *TMC1*  *TMEM132E*  *TMIE*  *TMPRSS3*  *TMTC2*  *TNC*  *TPRN*  *TRIOBP*  *TRRAP**  *TSPEAR*  *USH1C**  *USP48*  *WBP2*  *WFS1**  *WHRN** | *ABHD12*  *ACTB*  *ACTG1**  *ADGRV1*  *ALMS1*  *ANLN*  *AP1B1*  *APOPT1*  *ARSG*  *ATP1A3*  *ATP6V0A4*  *ATP6V1B1*  *ATP6V1B2*  *BCAP31*  *BCS1L*  *BMP4*  *BSND*  *CACNA1D*  *CDH23**  *CEP250*  *CEP78*  *CHD7*  *CIB2**  *CISD2*  *CLPP*  *CLRN1*  *COL11A1**  *COL11A2**  *COL2A1*  *COL4A3*  *COL4A4*  *COL4A5*  *COL9A1*  *COL9A2*  *COL9A3*  *DIAPH3*  *DMXL2**  *EDN3*  *EDNRB*  *EFNB2*  *ERAL1*  *ESPN**  *EXOSC2*  *EYA1*  *EYA4**  *FGF3*  *FITM2*  *FOXI1*  *GATA3*  *GPR98*  *GPRASP2*  *GPSM2**  *GRHL2**  *HARS*  *HARS2*  *HSD17B4*  *KARS**  *KCNE1*  *KCNJ10*  *KCNQ1*  *KIAA0391*  *KITLG**  *LARS2*  *LOXL3*  *MITF*  *MYH1*  *MYH14**  *MYH9**  *MYO6**  *MYO7A**  *NARS2**  *NDP*  *NLRP3**  *OPA1*  *PAX3*  *PCDH15**  *PET100*  *PEX1*  *PEX26*  *PEX6*  *PLOD3*  *PNPT1**  *POLR1C*  *POLR1D*  *POLR1C*  *POLR1D*  *POLD1*  *PRORP*  *PRPS1**  *RAI1*  *ROBO1*  *RRM2B*  *SEMA3A*  *SIX1**  *SIX5*  *SLC12A1*  *SLC12A2**  *SLC19A2*  *SLC25A2*  *SLC26A4**  *SLC29A3*  *SLC33A1*  *SLC52A2*  *SLC9A1*  *SLITRK6*  *SMOC1*  *SNAI2*  *SOX10*  *SPATA5*  *TANGO1*  *TBC1D24**  *TBL1Y*  *TCOF1*  *TIM88A*  *TIMM8A*  *TRRAP**  *TWNK*  *TYR*  *USH1C**  *USH1G*  *USH2A*  *WFS1**  *WHRN**  *YAP1* | *AK2*  *ALG11*  *ALG12*  *AMER1*  *ANKH*  *ANOS1*  *AP1S1*  *ASPA*  *ATP1A2*  *ATRX*  *AUTS2*  *BRAF*  *BTD*  *CARS2*  *CASK*  *CD151*  *CDK5RAP2*  *CEP57*  *CEP89*  *CHSY1*  *CLCNKA*  *CLCNKB*  *COL1A2*  *COQ6*  *COX10*  *CSPP1*  *CTSA*  *DCAF17*  *DCHS1*  *DIO2*  *DLD*  *DLX5*  *DNAAF3*  *DNMT1*  *DSTYK*  *DUSP6*  *ELAC2*  *ERCC3*  *ERCC4*  *FGF8*  *FGFR1*  *FGFR2*  *FGFR3*  *FKBP14*  *FLRT3*  *FOXC1*  *G6PC3*  *GALE*  *GATA2*  *GJA1*  *GJC3*  *GMPPB*  *HDAC8*  *HGSNAT*  *HOXA1*  *HOXA2*  *IARS2*  *IDUA*  *IGF1*  *IL17RD*  *INF2*  *IRX5*  *ITM2B*  *KIT*  *KRIT1*  *LHX3*  *LRP2*  *MAF*  *MANBA*  *MARS2*  *MBTPS2*  *MFN2*  *MGAT2*  *MKKS*  *MPZ*  *NAGLU*  *NDRG1*  *NDUFA9*  *NIPBL*  *NLRP12*  *NMNAT1*  *NOTCH3*  *OFD1*  *ORC1*  *PAX2*  *PCNA*  *PDSS1*  *PEX11B*  *PEX7*  *PHYH*  *PIGV*  *PMP22*  *PNPLA2*  *POLG*  *POMK*  *PRKAR1A*  *PROK2*  *PROKR2*  *PTRH2*  *RAD21*  *RFT1*  *RMND1*  *RPGR*  *RPS6KA3*  *RUNX2*  *SALL1*  *SALL4*  *SERAC1*  *SLC4A11*  *SLC52A3*  *SMAD4*  *SOX2*  *SPRY4*  *SPTBN4*  *SRP72*  *ST3GAL5*  *SUCLA2*  *TBL1X*  *TBX1*  *TCF12*  *THRB*  *TNFRSF11B*  *TPM2*  *TRPV4*  *TTR*  *UBR1*  *XPNPEP3*  *XYLT2* | *AADAT*  *AAK1*  *AARS*  *ABCA2*  *ABCB1*  *ABCD2*  *ABCD4*  *ABL2*  *ACAN*  *ACD*  *ACHE*  *ACSL4*  *ACVR2A*  *ADGRB1^#^*  *ADRA1D*  *ADRA2C*  *AEBP2*  *AFAP1L2*  *AFF1*  *AFF3*  *AFM*  *AGA*  *AGAP1^#^*  *AGTPBP1*  *AGTR2*  *AHSG*  *ALDH1A2*  *ALDH2*  *ALDH3A2*  *ALG10B*  *ANK1*  *ANK2*  *ANKRD11*  *ANP32B*  *AP180*  *AP3D1*  *AP3M2*  *AP3S1*  *AP4E1*  *APAF1*  *APOE*  *AQP4*  *ARHGAP33*  *ARSA*  *ASIC2*  *ATF2*  *ATF7*  *ATG4B*  *ATG5*  *ATP2B1*  *ATP7A*  *ATP8A2*  *ATP8B1*  *AXIN1*  *B3GALT2*  *B9D1*  *BACE1*  *BAIAP2L2*  *BARHL1^#^*  *BBS1*  *BBS2*  *BBS4*  *BCL2*  *BDNF*  *BHLHE40^#^*  *BLOC1S4*  *BLOC1S5*  *BLOC1S6*  *BMP5*  *BMP7*  *BMPER*  *BRD2^#^*  *BRD4*  *BRE*  *BRPF1*  *BSN*  *BSND*  *BSPRY*  *BTBD9*  *C12orf4^#^*  *C1QBP*  *C9ORF43*  *CA8*  *CACHD1*  *CACNA1A*  *CACNA1B*  *CACNA1H*  *CACNA2D3*  *CACNB2*  *CACNB4*  *CACNG2*  *CALCA*  *CAMLG*  *CAMSAP3^#^*  *CAPN5*  *CAPRIN1*  *CASP3*  *CBL*  *CBY1*  *CCDC88C*  *CCDC92*  *CDH11*  *CDK14^#^*  *CDKN1B*  *CDKN2D*  *CDO1*  *CELSR1^#^*  *CERS1*  *CGA*  *CHN1*  *CHRD*  *CHRM1*  *CHRNA10*  *CHRNA9^#^*  *CHRNB2*  *CHRNB3*  *CKB*  *CKMT1B*  *CLDN11*  *CLEC16A*  *CLP1*  *CNTN5*  *CNTNAP1*  *COL1A1*  *COLCA2*  *CPLX1*  *CPLX2*  *CRKL*  *CS*  *CSF1*  *CSK*  *CSNK1G3*  *CSNK2A1*  *CTCF*  *CTNNA2*  *CTNNB1*  *CTSF*  *CTSL*  *CXCR2^#^*  *CYB5R2*  *CYBA*  *CYP19A1*  *CYS1^#^*  *DAB1*  *DACH1*  *DCLK1*  *DDR1*  *DIO3*  *DISC1*  *DLG4*  *DLX1*  *DLX2*  *DLX6*  *DMD*  *DNAH5*  *DNAJC5*  *DNASE1*  *DNM1*  *DNM3*  *DRD1*  *DRD2*  *DRD3*  *DSCAM*  *DST*  *DUOX2*  *DUOXA2*  *DUSP1*  *DUSP7*  *DVL1*  *DVL3*  *DYNC1LI1*  *E2F4*  *ECE1*  *EDARADD*  *EDN1*  *EDNRA*  *EFNB1*  *EGFR*  *EGR2*  *EHMT1*  *EIF3C*  *ELMOD1*  *EMB*  *EMX1*  *EMX2*  *ENG*  *ENPP1*  *EPHA4*  *EPHB1*  *EPHB2*  *EPHB3*  *EPM2A*  *EPS8L1*  *ERBB2*  *ERBB4*  *ERCC6*  *ESR2*  *ESRRG*  *EWSR1*  *FADS3*  *FAM107B*  *FAM117B*  *FAM20C*  *FAM83G*  *FAS*  *FAT4*  *FBLN1*  *FBXO11*  *FBXO2^#^*  *FBXO33^#^*  *FGF10*  *FGF11*  *FGF12*  *FGF14*  *FGF20*  *FGF9*  *FGFR2*  *FGFR3*  *FIGN*  *FMR1*  *FOLR1*  *FOS*  *FOXC2*  *FOXG1*  *FOXI3*  *FOXO3*  *FOXP2*  *FOXP3*  *FREM2*  *FSCN2*  *FUZ*  *FYN*  *FZD4*  *GABBR1*  *GABRA1*  *GABRA3*  *GABRA5^#^*  *GABRB2*  *GABRB3*  *GAS1*  *GAS2L2^#^*  *GBX2*  *GDF6*  *GFI1*  *GFRA1*  *GGA1*  *GLI2*  *GLI3*  *GLRA1*  *GLRB*  *GMFB*  *GNA11*  *GNAO1*  *GNAQ*  *GNAS*  *GNG3*  *GNG7*  *GPR126*  *GPR152^#^*  *GPR50*  *GPX1*  *GRB2*  *GRIA3*  *GRIA4*  *GRID1^#^*  *GRID2*  *GRIK5*  *GRIN1*  *GRIN2A*  *GRIN2B*  *GRIN2C*  *GRIN2D*  *GRM1*  *GSC*  *GSTA3*  *GUSB*  *HAND2*  *HBP1*  *HCN2*  *HERC1*  *HES1*  *HES5*  *HESX1*  *HIC1*  *HMGA2*  *HMX1*  *HMX2*  *HMX3^#^*  *HOXA5*  *HOXB1*  *HOXB2*  *HPN*  *HPS1*  *HPS4*  *HPS6*  *HR*  *HSD17B2*  *HSPG2*  *HTR1B*  *HTRA2*  *HTT*  *IFITM1*  *IFT88*  *IGF1R*  *IKZF2^#^*  *IKZF5*  *IL1R2*  *IREB2*  *IRF6*  *IRS1*  *ISL1*  *ITGA8*  *ITPR1*  *JAG1*  *JAG2*  *JAK1*  *KALRN*  *KAT6B*  *KCNA1*  *KCNH5*  *KCNJ12*  *KCNJ16*  *KCNJ6*  *KCNMA1*  *KDF1*  *KDM8*  *KIF14*  *KIF1A*  *KIF27*  *KL*  *KLC2*  *KLHL18^#^*  *KMT2A*  *KRT10*  *KRT2*  *LAMA2*  *LAMA4*  *LAMC2*  *LARGE*  *LATS1*  *LATS2*  *LEPRE1*  *LFNG*  *LGI1*  *LIMK2*  *LMNA*  *LMNB2*  *LMO4*  *LMO7*  *LRIG1^#^*  *LRIG2*  *LRIG3*  *LRP6*  *LRRC4^#^*  *LSM1*  *LTN1*  *LYST*  *MAFB*  *MAG*  *MAN2B1*  *MAOA*  *MAP1A*  *MAP3K1^#^*  *MAPT*  *MARC2*  *MARCH9*  *MAT2A*  *MBP*  *MCOLN3*  *MCPH1*  *MDK*  *MECOM*  *MECP2*  *MED28*  *MIB2*  *MID1*  *MIF^#^*  *MINAR2*  *MIR122*  *MIR182*  *MKRN2^#^*  *MKS1*  *MMP14*  *MOS*  *MPDZ*  *MPV17*  *MRO*  *MSX1*  *MSX2*  *MYCN*  *MYD88*  *MYH10*  *MYO1A*  *MYO1C*  *MYO5A*  *MYSM1*  *NABP2*  *NAPA*  *NAV2*  *NDST1*  *NDUFS4*  *NEDD4L*  *NEU1*  *NEUROD1*  *NEUROD4*  *NEUROG1*  *NFATC3*  *NFIX*  *NFKB1*  *NGFR*  *NIN*  *NISCH*  *NKX3-2*  *NLGN3*  *NOG*  *NOS1*  *NOTCH1*  *NOX3*  *NOXO1*  *NPAS3*  *NPC1*  *NPR2*  *NPTN^#^*  *NR2F1*  *NR4A3*  *NRP1*  *NRTN*  *NTF3*  *NTF4*  *NTN1*  *NTNG2^#^*  *NTRK2*  *NTRK3*  *NTSR1*  *NTSR2*  *NUP88*  *NXPH3*  *OC90^#^*  *OCA2*  *OCM^#^*  *ODF3L2*  *OPRL1*  *OSR2*  *OTOP1*  *OTOP3*  *OTOS^#^*  *OTUD7B*  *OTULIN*  *OTX1^#^*  *OTX2*  *OVOL2*  *OXGR1*  *P2RX7^#^*  *PACS2*  *PAH*  *PARK2*  *PARL*  *PARP1*  *PAX6*  *PAX8*  *PAX9*  *PBX1*  *PEPD*  *PEX2*  *PEX3^#^*  *PEX5L*  *PGAP1*  *PHEX*  *PHF20^#^*  *PHF6*  *PHOX2A*  *PHOX2B*  *PHYKPL*  *PIP5K1C*  *PKD1*  *PKHD1L1*  *PLAU*  *PLCB1*  *PLP1*  *PNOC*  *POLH*  *POU1F1*  *POU3F3*  *POU4F1*  *PPARGC1A*  *PPM1A*  *PPP2R5D*  *PREP*  *PREX2*  *PRKRA*  *PRODH*  *PROP1*  *PRRX1*  *PSAP*  *PSEN1*  *PSTPIP2*  *PTCH1*  *PTGER1*  *PTK7*  *PTN*  *PTPN11*  *PTPRG*  *PVRL1*  *PVRL3*  *RARA*  *RASAL2*  *RBFOX3*  *RBM24*  *RBPJ*  *RDH10*  *RELN*  *RERE*  *RGN*  *RLN3*  *RND3*  *RNF103*  *ROBO3*  *ROR2*  *RORA*  *RPL27A*  *RPL38*  *RSAD1*  *RSF7*  *SCARB2*  *SCN8A*  *SCRIB*  *SCUBE1*  *SEC24B*  *SELENOK*  *SEMA3F^#^*  *SEMA5B*  *SEPT5*  *SETD5^#^*  *SFN*  *SFSWAP*  *SFXN3*  *SGCE*  *SH3PXD2B*  *SHANK3*  *SHH*  *SLC12A6*  *SLC12A7^#^*  *SLC16A10*  *SLC16A2*  *SLC17A5*  *SLC17A7*  *SLC17A8*  *SLC19A2*  *SLC1A3*  *SLC24A5*  *SLC25A21*  *SLC2A3*  *SLC30A4*  *SLC38A10*  *SLC4A10*  *SLC4A7*  *SLC5A5*  *SLC6A1*  *SMARCA4*  *SMS*  *SNAP25*  *SOBP^#^*  *SOD1*  *SOD2*  *SOX9*  *SP4*  *SPG7*  *SPRY2*  *SPTBN1*  *SPTBN2*  *SRR*  *SRRM4^#^*  *STK36*  *STRN*  *STUB1*  *STX1A*  *SUN1^#^*  *SYNGAP1*  *SYNJ2*  *TBC1D2B*  *TBCE*  *TBX10*  *TBX15*  *TBX18*  *TCF15*  *TCF4*  *TECTB^#^*  *TFAP2A*  *TGFA*  *TGFB2*  *TGIF1*  *THRA*  *TMC2*  *TMEM30B*  *TMEM51*  *TMOD2*  *TMTC4*  *TNF*  *TNFRSF1B*  *TOR1A*  *TOX*  *TP73*  *TPO*  *TPP1*  *TRAM2^#^*  *TRPC3^#^*  *TRPML3*  *TSHR*  *TSHZ1*  *TSPAN12*  *TTLL1*  *TTLL4*  *TUB*  *TUBA1A*  *TWIST2*  *TWSG1*  *TYROBP*  *TYRP1*  *UBE2B*  *UBE2G1*  *UBE2W*  *UBE3A*  *UBE3B*  *UCN^#^*  *UCP1*  *ULK4*  *USP42^#^*  *VANGL1*  *VANGL2*  *VDR*  *VIM*  *VTI1A*  *WASF1*  *WDPCP*  *WDR19*  *WDTC1*  *WNT1*  *WNT5A*  *WTAP*  *XBP1*  *XYLB*  *YWHAE*  *ZCCHC14*  *ZEB1*  *ZIC2*  *ZIC3*  *ZNF175^#^* | *ABCA13*  *ABCB5*  *ACSM6*  *ACSS3*  *ADAM20*  *ADAMTSL3*  *AHCYL2*  *AIF1L*  *AKAP9*  *AKNAD1*  *ALOX15*  *ANKRD31*  *ANKRD36C*  *ANP32A*  *ANP32D*  *APOD*  *APOL2*  *APOOL*  *ARF3*  *ARHGAP10*  *ARID4A*  *ARL17A*  *ARMC4*  *ARR3*  *ASH1L*  *ATP13A5*  *BAZ1B*  *BLID*  *BMP5*  *BOD1L1*  *BPTF*  *BRIP1*  *BTF3L4*  *C10orf12*  *C15orf32*  *C15orf54*  *C19orf68*  *C1orf195*  *C1QTNF1-AS1*  *C2orf49*  *C6orf10*  *C8orf34*  *CA3*  *CBX5*  *CCDC144A*  *CCDC148*  *CCDC152*  *CCDC186*  *CCDC81*  *CEMIP*  *CEP128*  *CHRNA6*  *CHRNA9*  *CHRNB4*  *CIB3*  *CLDN19*  *CNTF*  *CNTLN*  *COL10A1*  *COLEC10*  *CP*  *CREB3L2*  *CSNK2A3*  *CWF19L2*  *CYP1B1*  *DAZL*  *DCDC2C*  *DCT*  *DDR2*  *DDX43*  *DEFB108B*  *DNAJC5B*  *DYNAP*  *DYTN*  *EBF1*  *EBLN2*  *EDDM3B*  *EFCAB6*  *EGF*  *EHBP1*  *EIF5B*  *ENPP6*  *EPYC*  *ESF1*  *EYA2*  *F2RL2*  *FAM19A3*  *FAT4*  *FBXL20*  *FGFBP3*  *FIP1L1*  *FKBP2*  *FMN1*  *FOXD1*  *FOXO3*  *FRK*  *FRS2*  *FRZB*  *FSBP*  *FSIP2*  *GAS2L3*  *GCC2*  *GCNT7*  *GDF11*  *GLCE*  *GLIPR1L1*  *GOLGA4*  *GOLGB1*  *GOLIM4*  *GPHA2*  *GPX6*  *HBD*  *HCAR1*  *HHLA1*  *IFNK*  *IGSF6*  *IQCJ*  *IQUB*  *ITGA10*  *ITSN1*  *KCNA10*  *KCNJ13*  *KIAA0754*  *KIAA1024L*  *KIAA1217*  *KLHL31*  *KRTAP26-1*  *LANCL3*  *LECT1*  *LEMD1*  *LEO1*  *LHCGR*  *LHFP*  *LMO7DN*  *LRRC38*  *LRRN1*  *MAML2*  *MAX*  *MED31*  *METTL5*  *MFAP1*  *MIA*  *MKX*  *MLANA*  *MLNR*  *MME*  *MMP21*  *MNS1*  *MPHOSPH8*  *MPP6*  *MRAS*  *MTRNR2L1*  *MTRNR2L10*  *MTRNR2L2*  *MTRNR2L6*  *MTRNR2L8*  *MTRNR2L9*  *MUC6*  *MYH8*  *MYLK4*  *MYO3B*  *MYRFL*  *NACA2*  *NBEAL1*  *NCL*  *NCMAP*  *NDNF*  *NDST4*  *NET1*  *NFAT5*  *NFIA*  *NHLRC2*  *NKX6-1*  *NLRP14*  *NME7*  *NOTCH2NL*  *NOX3*  *NPM1*  *NPR3*  *NSRP1*  *OC90*  *OCLM*  *OCM*  *OCM2*  *OR10AD1*  *OR1C1*  *OR2AE1*  *OR2D3*  *OR51I1*  *OR51M1*  *OR51Q1*  *OR52B6*  *OR6V1*  *OTOL1*  *OTOR*  *OXTR*  *PALM3*  *PATE1*  *PATE3*  *PBOV1*  *PCDHB11*  *PCDHB12*  *PCDHB5*  *PCDHB7*  *PCDHGA2*  *PCDHGA3*  *PCDHGB1*  *PCDHGB3*  *PCM1*  *PDCD10*  *PDK3*  *PGAP1*  *PHACTR2*  *PHF3*  *PHKA1*  *PHLDB2*  *PIAS1*  *PITPNB*  *PLA2G4A*  *PLCB4*  *PLEKHA3*  *PLEKHA4*  *PLET1*  *POLR3G*  *POU5F2*  *PP2D1*  *PPBP*  *PPFIBP1*  *PPIG*  *PPIL4*  *PREX2*  *PRPF38B*  *PRPSAP1*  *PRSS48*  *PSG6*  *PTN*  *PTX3*  *RAB7A*  *RARRES1*  *RBM25*  *RBM41*  *RELL1*  *RGPD1*  *RGS22*  *RNF152*  *RNF214*  *RSF1*  *S100A13*  *S100B*  *SAMD15*  *SCAF11*  *SEMA3D*  *SEMA3E*  *SETBP1*  *SH2D4B*  *SHPRH*  *SIAH1*  *SLC13A4*  *SLC16A4*  *SLC20A2*  *SLC22A2*  *SLC6A20*  *SLC9A4*  *SLCO1C1*  *SMARCC1*  *SMC3*  *SMCO3*  *SMG7*  *SNX2*  *SPATA17*  *SPIN2A*  *SPX*  *SSX1*  *ST3GAL6*  *ST8SIA2*  *STC2*  *STK39*  *STT3A*  *SYCP3*  *TAS2R13*  *TAS2R3*  *TAS2R30*  *TAS2R46*  *TAS2R50*  *TAS2R9*  *TBX18*  *TDRD1*  *TECTB*  *TEDDM1*  *TET1*  *TFDP1*  *TGFB2*  *TIMM23B*  *TJP1*  *TMEM14EP*  *TMOD1*  *TMPPE*  *TMPRSS11D*  *TPR*  *TRAF3IP1*  *TRAPPC3L*  *TRAPPC9*  *TRIP11*  *TROVE2*  *TRPM3*  *UACA*  *UCMA*  *UHRF2*  *UPF3A*  *USP17L2*  *USP53*  *UTRN*  *VEPH1*  *WFIKKN2*  *YIPF6*  *ZBTB20*  *ZC3H13*  *ZC3H6*  *ZFHX3*  *ZFHX4*  *ZNF292*  *ZNF460*  *ZNF462*  *ZNF483*  *ZNF788*  *ZNHIT6*  *ZP2* | *A2M*  *AASDH*  *ABCA1*  *ABCE1*  *ACADM*  *ACAT2*  *ACSL3*  *ADAMTS9*  *ADAR*  *ADI1*  *ADNP*  *ADPRM*  *AHCYL2*  *AHNAK*  *AHNAK2*  *AKAP11*  *AKR1B1*  *ALDH1A1*  *ALDH9A1*  *AMOTL1*  *ANAPC5*  *ANGPT1*  *ANK2*  *ANKRD10*  *ANO6*  *ANP32A*  *ANP32B*  *ANTXR1*  *ANXA2*  *AP1S2*  *API5*  *APLP2*  *APMAP*  *APOD*  *APOO*  *APP*  *APPBP2*  *ARHGAP15*  *ARHGAP5*  *ARHGEF26*  *ARHGEF4*  *ARID1A*  *ARID1B*  *ARL5B*  *ARL6IP5*  *ARMC9*  *ARPC2*  *ARPC3*  *ASAH1*  *ASPN*  *ATF4*  *ATP1B3*  *ATP2A2*  *ATP2B4*  *ATP5A1*  *ATP5B*  *ATP5G3*  *ATP5J*  *ATP6V0C*  *ATP6V0E1*  *ATP6V1A*  *BAIAP2*  *BAIAP2L1*  *BAMBI*  *BBS2*  *BCHE*  *BHLHE40*  *BMP5*  *BOC*  *BRD4*  *BRD8*  *BTG1*  *BTG2*  *BTG3*  *BUB3*  *BUD31*  *BVES*  *BZW2*  *C11orf48*  *C11orf53*  *C14orf119*  *C14orf166*  *C16orf72*  *C17orf75*  *C1QBP*  *C1QTNF3*  *C20orf194*  *C2orf40*  *C3AR1*  *C3orf62*  *C5orf15*  *C6orf62*  *C7*  *C7orf60*  *CA3*  *CACHD1*  *CACYBP*  *CADM1*  *CADPS2*  *CALCOCO2*  *CALM1*  *CALM2*  *CALU*  *CASC4*  *CAT*  *CBX3*  *CCDC8*  *CCDC80*  *CCNI*  *CCT3*  *CCT5*  *CCT8*  *CD24*  *CD302*  *CD63*  *CD74*  *CD9*  *CD93*  *CDC14B*  *CDC42*  *CDC5L*  *CDH11*  *CDKN1A*  *CDKN1B*  *CDKN1C*  *CDS2*  *CEBPD*  *CEMIP*  *CENPJ*  *CERS2*  *CFL1*  *CGA*  *CHCHD2*  *CHCHD3*  *CHD9*  *CHST11*  *CIR1*  *CIRBP*  *CKS2*  *CLASP2*  *CLDN19*  *CLEC1A*  *CLK1*  *CLMP*  *CLNS1A*  *CLPTM1*  *CLSTN1*  *CLTC*  *CLU*  *CMPK1*  *CNBD2*  *CNDP2*  *CNKSR3*  *CNN3*  *CNOT1*  *CNRIP1*  *CNTN1*  *CNTN6*  *COL1A1*  *COL27A1*  *COL3A1*  *COL4A1*  *COL4A2*  *COL5A2*  *COL6A1*  *COL6A2*  *COL8A1*  *COLEC12*  *COPA*  *COPS5*  *COQ10B*  *COTL1*  *COX4I1*  *COX5A*  *COX6B1*  *COX7B*  *COX7B2*  *COX7C*  *CPE*  *CPNE2*  *CPXM1*  *CRABP2*  *CREBBP*  *CRISPLD1*  *CRISPLD2*  *CRYBA1*  *CSE1L*  *CSF1R*  *CSH2*  *CSMD3*  *CSRNP1*  *CSRNP2*  *CST3*  *CTBP1*  *CTBP2*  *CTGF*  *CTHRC1*  *CTNNA1*  *CTNNAL1*  *CTNNB1*  *CTNNBIP1*  *CTNND1*  *CTSE*  *CTSK*  *CUL1*  *CWC15*  *CYFIP1*  *CYP1B1*  *CYP24A1*  *DAAM2*  *DAB1*  *DAB2*  *DCAF16*  *DCAF7*  *DCN*  *DCP2*  *DCTN2*  *DDX17*  *DDX3X*  *DDX5*  *DDX50*  *DENND6A*  *DERL1*  *DGCR6*  *DGKI*  *DHRS3*  *DHX15*  *DHX30*  *DICER1*  *DKFZp586I1420*  *DLL1*  *DMTF1*  *DNAJA2*  *DNAJB11*  *DNAJC1*  *DNAJC8*  *DNAJC9*  *DOCK3*  *DST*  *DTL*  *DUSP1*  *DUSP14*  *DUT*  *DYNC1I1*  *DYNC1I2*  *DYNLL1*  *DZIP1*  *EBF1*  *EDEM3*  *EDNRA*  *EEF1A1*  *EEF1B2*  *EEF1E1*  *EEF1G*  *EEF2*  *EGFL6*  *EIF1*  *EIF2AK2*  *EIF3A*  *EIF4A2*  *EIF4E*  *EIF4G2*  *ELF2*  *ELK3*  *ELN*  *EMC2*  *EMCN*  *ENAH*  *ENPP1*  *EPB41L1*  *EPB41L3*  *EPCAM*  *EPHA5*  *EPHA7*  *EPYC*  *ERLEC1*  *ERP44*  *ESD*  *ETF1*  *ETNK1*  *ETS2*  *EWSR1*  *EXD2*  *EXOSC10*  *EXTL3*  *EYA3*  *F3*  *FAM102A*  *FAM110B*  *FAM120A*  *FAM126B*  *FAM160B1*  *FAM217B*  *FAM32A*  *FAM45A*  *FAM49B*  *FARP1*  *FAT4*  *FAU*  *FBL*  *FBLN2*  *FBXO33*  *FCHO2*  *FKBP6*  *FKBP9*  *FLI1*  *FLNB*  *FLRT2*  *FMO1*  *FN1*  *FNBP4*  *FNDC3A*  *FNTA*  *FOCAD*  *FOS*  *FOXC2*  *FOXD3*  *FOXO3*  *FOXP2*  *FRMD8*  *FRZB*  *FSTL1*  *FTH1*  *FTL*  *FTO*  *FUS*  *FXYD6*  *FZD1*  *FZD3*  *GALNT10*  *GALNT2*  *GAPDH*  *GARNL3*  *GCFC2*  *GCNT4*  *GDF10*  *GH1*  *GID8*  *GLE1*  *GLI3*  *GLO1*  *GLRX2*  *GLTSCR1L*  *GLUL*  *GMPR2*  *GNAI3*  *GNAS*  *GNB2L1*  *GNB4*  *GNG10*  *GNG12*  *GNG2*  *GNG5*  *GNPDA1*  *GNS*  *GOLGA5*  *GOLM1*  *GPAM*  *GPC4*  *GPC6*  *GPR125*  *GPRC5B*  *GRB2*  *GRIK4*  *GRN*  *GSN*  *GSPT1*  *GTF2B*  *GTF2H1*  *GTF3A*  *GUK1*  *GYG1*  *H2AFY*  *H3F3B*  *HAT1*  *HBB*  *HBE1*  *HBG1*  *HBG2*  *HBP1*  *HCFC1*  *HCRTR1*  *HDAC1*  *HDAC2*  *HDGFRP3*  *HDHD2*  *HEPH*  *HLA-DRA*  *HMCN1*  *HMGN1*  *HMGN2*  *HMGN3*  *HNRNPA1*  *HNRNPA2B1*  *HNRNPA3*  *HNRNPA3P1*  *HNRNPC*  *HNRNPH1*  *HNRNPH2*  *HNRNPR*  *HNRNPU*  *HP1BP3*  *HPCAL1*  *HSBP1*  *HSD17B7*  *HSP90AA1*  *HSP90AB1*  *HSP90B1*  *HSPA1A*  *HSPA4L*  *HSPA9*  *HSPD1*  *HTRA1*  *HTRA3*  *IARS*  *IBSP*  *ICE2*  *ICMT*  *ID1*  *ID3*  *IFIT1*  *IFITM2*  *IFITM3*  *IFRD1*  *IGBP1*  *IGF2*  *IGFBP5*  *IGSF10*  *IL10RB*  *IL6ST*  *ILF2*  *ILF3*  *IMP3*  *IMPDH2*  *IQCK*  *IQSEC1*  *IRF2BP2*  *IRS2*  *IRX2*  *IST1*  *ITGA6*  *ITGA8*  *ITM2A*  *ITM2C*  *JAM3*  *JTB*  *JUN*  *KCNJ5*  *KCTD12*  *KCTD6*  *KDELR1*  *KDM2B*  *KDM3B*  *KDM5B*  *KHDRBS1*  *KIDINS220*  *KIF13B*  *KIF1A*  *KLHDC2*  *KLHL15*  *KLHL21*  *KLHL23*  *KMT2C*  *LAMA2*  *LAMB1*  *LAMC1*  *LAPTM4A*  *LATS2*  *LCMT1*  *LDHB*  *LEAP2*  *LECT1*  *LEF1*  *LGALS1*  *LHFP*  *LIPA*  *LITAF*  *LMBRD1*  *LMO7*  *LNX1*  *LPAR1*  *LPIN1*  *LPIN2*  *LRBA*  *LRCH1*  *LRP1B*  *LRPPRC*  *LRRC49*  *LRRN1*  *LSM8*  *LSS*  *LYPD6*  *LYVE1*  *MAEA*  *MAFB*  *MAGED1*  *MAGED2*  *MAN1A1*  *MANF*  *MAP2K1*  *MAP3K1*  *MAP3K7*  *MAP4K3*  *MAP4K4*  *MAPK9*  *MAPKAPK2*  *MAPRE1*  *MARCKS*  *MATN2*  *MBD2*  *MBOAT2*  *MBP*  *MBTPS1*  *MCAM*  *MCL1*  *MDH1*  *MECOM*  *MED1*  *MED26*  *MEIS3P1*  *MFNG*  *MIA*  *MIEF1*  *MIER3*  *MKL2*  *MKLN1*  *MLANA*  *MMP13*  *MOB1A*  *MON2*  *MORC3*  *MORF4L1*  *MPV17*  *MPZL1*  *MRFAP1*  *MRFAP1L1*  *MRPL20*  *MRPL42*  *MRPL49*  *MRPL51*  *MRPL9*  *MRPS21*  *MRPS34*  *MRPS6*  *MS4A7*  *MST1*  *MTCL1*  *MTPN*  *MUM1*  *MVD*  *MYCBP2*  *MYH10*  *MYL12B*  *MYLPF*  *MYRIP*  *NAALAD2*  *NAB1*  *NACA*  *NAP1L1*  *NAP1L4*  *NBEAL1*  *NCAM1*  *NCBP2*  *NCL*  *NDNF*  *NDUFB2*  *NDUFC2*  *NDUFS5*  *NECAP1*  *NEFL*  *NHP2L1*  *NID1*  *NID2*  *NKTR*  *NOA1*  *NOB1*  *NONO*  *NOTCH2*  *NOV*  *NPC2*  *NPTN*  *NR1D1*  *NR2C1*  *NR2F1*  *NREP*  *NSF*  *NSMAF*  *NSMCE1*  *NTRK2*  *NUCKS1*  *NUDT16*  *NUP107*  *NXF1*  *NXN*  *NXPH1*  *OAZ1*  *OAZ2*  *OCIAD2*  *OGN*  *OLFM1*  *OLFML2A*  *OR7E14P*  *ORC3*  *OS9*  *OSMR*  *OSTC*  *OTOR*  *OXA1L*  *P4HA1*  *P4HA3*  *P4HB*  *PABPC4*  *PAK2*  *PAM*  *PAN3*  *PAPD4*  *PAPOLA*  *PARP1*  *PCDH18*  *PCDHGC5*  *PCNP*  *PCNT*  *PCSK4*  *PCSK5*  *PCYT2*  *PDCD6IP*  *PDE11A*  *PDE1A*  *PDE4D*  *PDGFRA*  *PDGFRL*  *PDIA4*  *PDIA6*  *PDZD2*  *PDZK1*  *PEAK1*  *PEBP1*  *PEG10*  *PEX19*  *PEX3*  *PGAM1*  *PGK1*  *PGM1*  *PHACTR2*  *PHB2*  *PHKB*  *PHLDB2*  *PID1*  *PIGF*  *PIGG*  *PIGK*  *PIGY*  *PIK3AP1*  *PIK3C2A*  *PIK3C3*  *PIP5K1B*  *PKD1*  *PLAG1*  *PLAT*  *PLD5*  *PLEKHA1*  *PLOD1*  *PLP1*  *PLSCR4*  *PLTP*  *PLXNA2*  *PMEPA1*  *PNRC1*  *POLR2L*  *POSTN*  *PPA1*  *PPBP*  *PPDPF*  *PPIA*  *PPP1R12B*  *PPP1R1C*  *PPP2CA*  *PPP2CB*  *PPP2R3A*  *PRCP*  *PRDM16*  *PRDX4*  *PRDX6*  *PRICKLE4*  *PRKAB2*  *PRKCI*  *PRKD3*  *PRKRA*  *PRMT3*  *PRMT8*  *PRNP*  *PROS1*  *PRPS2*  *PRRC2B*  *PRSS12*  *PRSS35*  *PRUNE*  *PSAP*  *PSMB1*  *PSMB5*  *PSMC1*  *PSMC2*  *PSMC3IP*  *PSMC6*  *PSMD12*  *PTBP1*  *PTBP2*  *PTCH1*  *PTGER4*  *PTGES3*  *PTGIS*  *PTGS1*  *PTGS2*  *PTK2*  *PTMA*  *PTN*  *PTPN11*  *PTPRG*  *PTTG1IP*  *PUM1*  *PURA*  *PYCR2*  *RAB1A*  *RAB7A*  *RABL2B*  *RAC1*  *RAD51C*  *RAN*  *RANBP9*  *RAP1GDS1*  *RAPH1*  *RBM23*  *RBMS1*  *RCBTB2*  *RCN1*  *RECQL5*  *REEP5*  *RELN*  *RESP18*  *RFX5*  *RGS3*  *RGS5*  *RHBDF1*  *RHOA*  *RHOBTB3*  *RIMS2*  *RIN2*  *RIPK1*  *RNF11*  *RNF130*  *RNF168*  *RNF19A*  *RNPS1*  *ROMO1*  *RPA1*  *RPA3*  *RPL10A*  *RPL11*  *RPL12*  *RPL13*  *RPL13A*  *RPL15*  *RPL19*  *RPL23*  *RPL26L1*  *RPL27*  *RPL27A*  *RPL29*  *RPL30*  *RPL31*  *RPL32*  *RPL35*  *RPL35A*  *RPL37*  *RPL37A*  *RPL38*  *RPL39*  *RPL4*  *RPL41*  *RPL5*  *RPL6*  *RPL7A*  *RPL8*  *RPLP0*  *RPLP1*  *RPLP2*  *RPN2*  *RPS10*  *RPS11*  *RPS12*  *RPS13*  *RPS14*  *RPS15*  *RPS15A*  *RPS17*  *RPS18*  *RPS2*  *RPS21*  *RPS23*  *RPS24*  *RPS25*  *RPS27*  *RPS27A*  *RPS28*  *RPS29*  *RPS3*  *RPS3A*  *RPS4X*  *RPS5*  *RPS6*  *RPS8*  *RRAGC*  *RRBP1*  *RRM1*  *RSRC2*  *RSRP1*  *RTN4*  *S100A10*  *S100B*  *SAP130*  *SAP18*  *SASH1*  *SAT1*  *SCARB2*  *SCN7A*  *SCRIB*  *SCRN1*  *SDC2*  *SDCBP*  *SEC14L1*  *SEC23A*  *SEC61B*  *SEL1L*  *SELT*  *SEMA3C*  *SEMA3E*  *SEMA5A*  *SEMA6D*  *SEPP1*  *SEPT2*  *SEPT8*  *SERBP1*  *SERPINH1*  *SERPINI1*  *SERTAD4*  *SESN3*  *SET*  *SETD5*  *SFPQ*  *SFRP1*  *SFRP4*  *SFT2D1*  *SGK2*  *SH3BGRL3*  *SHISA5*  *SIAH1*  *SIK1*  *SIPA1L2*  *SKP1*  *SLC22A5*  *SLC24A3*  *SLC25A28*  *SLC25A38*  *SLC25A4*  *SLC25A5*  *SLC25A6*  *SLC27A1*  *SLC30A9*  *SLC35E1*  *SLC35F1*  *SLC41A3*  *SLC7A11*  *SMG5*  *SMOC2*  *SNAI1*  *SND1*  *SNRNP27*  *SNRPB2*  *SNRPC*  *SNRPG*  *SOD1*  *SORBS1*  *SP4*  *SP7*  *SPARC*  *SPARCL1*  *SPG21*  *SPINT2*  *SPP1*  *SPTBN1*  *SRI*  *SRM*  *SRP14*  *SRP9*  *SRPK1*  *SRPX*  *SRSF10*  *SRSF11*  *SRSF5*  *SS18*  *SSBP1*  *SSPN*  *ST13*  *STAT2*  *STAT6*  *STAU1*  *STMN1*  *STT3B*  *STUB1*  *STX3*  *SUB1*  *SUCLG1*  *SUFU*  *SULF2*  *SUMO2*  *SUMO3*  *SUN1*  *SUPT3H*  *SURF2*  *SURF4*  *SUSD1*  *SV2C*  *SYTL2*  *TAOK1*  *TARS*  *TBC1D5*  *TBL1XR1*  *TBX18*  *TCEAL1*  *TCF25*  *TCF3*  *TCFL5*  *TCP1*  *TCTN3*  *TERF2IP*  *TEX2*  *TFG*  *TFRC*  *TGFBR2*  *TGM2*  *TH*  *THBS2*  *THSD1*  *THSD4*  *TIA1*  *TIMM8B*  *TIMP2*  *TIMP3*  *TLE1*  *TLK1*  *TM4SF1*  *TMA7*  *TMBIM6*  *TMEM106A*  *TMEM131*  *TMEM133*  *TMEM147*  *TMEM167A*  *TMEM2*  *TMEM200B*  *TMEM258*  *TMEM263*  *TMEM51*  *TMPO*  *TMSB10*  *TMX1*  *TNIK*  *TOMM7*  *TPBG*  *TPM1*  *TPM3*  *TPP2*  *TPT1*  *TRA2B*  *TRAPPC11*  *TRAPPC8*  *TRIB2*  *TRIM2*  *TRIM33*  *TRIP6*  *TRPM3*  *TSC22D1*  *TSPAN3*  *TSPAN7*  *TSPYL1*  *TSPYL4*  *TSPYL5*  *TTC28*  *TTC3*  *TTC37*  *TTYH2*  *TUBA1B*  *TUBB*  *TUBB2A*  *TUG1*  *TUSC3*  *TWF1*  *TXNDC5*  *TXNIP*  *TYK2*  *TYROBP*  *U2SURP*  *UBA52*  *UBE2A*  *UBE2E2*  *UBE2E3*  *UBE2G1*  *UBE2N*  *UBE2Q1*  *UBE2V1*  *UBE3A*  *UBL3*  *UBR5*  *UGT2B10*  *UHRF1BP1L*  *UNC13B*  *UNC5C*  *UQCRB*  *UQCRH*  *UQCRQ*  *USMG5*  *USP33*  *USP37*  *VAPB*  *VASH2*  *VCL*  *VIM*  *VPS26A*  *VPS4A*  *VPS8*  *VRK1*  *WASF2*  *WBP1L*  *WBP5*  *WDFY3*  *WDR36*  *WDR47*  *WDR82*  *WIPF1*  *WISP1*  *WIZ*  *WRNIP1*  *WWTR1*  *XIST*  *XPNPEP1*  *XPO7*  *XRCC5*  *XRCC6*  *YBX1*  *YEATS2*  *YIPF2*  *YTHDC2*  *YWHAB*  *YWHAE*  *YWHAQ*  *ZACN*  *ZBTB33*  *ZBTB5*  *ZCCHC3*  *ZCCHC8*  *ZEB2*  *ZFAND5*  *ZFHX4*  *ZFP36L1*  *ZFP36L2*  *ZFP91*  *ZIC2*  *ZNF23*  *ZNF260*  *ZNF302*  *ZNF32*  *ZNF514*  *ZNF532*  *ZNF638*  *ZNHIT6*  *ZSCAN12*  *ZYG11B* | *A2M*  *AAAS*  *ABCA1*  *ABCA8*  *ABCA9*  *ABHD2*  *ABI1*  *ABTB2*  *ACACA*  *ACAD9*  *ACSL4*  *ACVR2A*  *ACYP2*  *ADAMTS12*  *ADAMTS4*  *ADAMTS9*  *ADARB1*  *ADD3*  *ADIPOR2*  *ADK*  *ADNP*  *AFF3*  *AGAP8*  *AGSK1*  *AHCYL2*  *AHI1*  *AKAP12*  *AMHR2*  *AMY1A*  *AMY1B*  *AMY1C*  *AMY2A*  *AMY2B*  *ANK2*  *ANK3*  *ANKFN1*  *ANKHD1*  *ANKHD1-EIF4EBP3*  *ANKIB1*  *ANKRD10*  *ANKRD17*  *ANKRD28*  *ANKRD36*  *ANKRD44*  *ANO2*  *ANTXR1*  *ANTXR2*  *ANXA2*  *ANXA2P2*  *AP3M2*  *APBB2*  *APOA1*  *APOA4*  *APOA5*  *APOC3*  *APP*  *ARGLU1*  *ARHGAP10*  *ARHGAP15*  *ARHGAP17*  *ARHGAP42*  *ARHGEF10*  *ARHGEF10L*  *ARHGEF12*  *ARID1A*  *ARID1B*  *ARID2*  *ARID5B*  *ARIH1*  *ARL15*  *ARPP19*  *ASAP1*  *ASAP2*  *ASCC1*  *ASTN2*  *ATAD1*  *ATAD2B*  *ATG7*  *ATL2*  *ATP2B4*  *ATP2C1*  *ATP6V1C1*  *ATP6V1G2-DDX39B*  *ATP8A1*  *ATP8B4*  *ATRNL1*  *ATXN1*  *ATXN2*  *B3GNT9*  *BACH2*  *BAG4*  *BAI3*  *BAZ1B*  *BBX*  *BCAS3*  *BCAT1*  *BCL10*  *BCL11A*  *BCL9*  *BCLAF1*  *BCYRN1*  *BICC1*  *BIRC6*  *BLCAP*  *BMP2K*  *BMPR1A*  *BMPR1B*  *BMPR2*  *BNC2*  *BNIP3L*  *BOC*  *BPTF*  *BRD8*  *BTBD11*  *BTBD7*  *BTBD9*  *BTN2A1*  *BUD13*  *C10orf11*  *C10orf46*  *C10orf68*  *C10orf76*  *C11orf67*  *C12orf10*  *C12orf39*  *C14orf28*  *C16orf70*  *C16orf72*  *C18orf25*  *C18orf8*  *C1orf21*  *C20orf118*  *C20orf132*  *C20orf194*  *C2CD3*  *C2orf63*  *C3orf23*  *C3orf70*  *C4orf21*  *C5orf22*  *C5orf45*  *C6orf170*  *C6orf211*  *C7orf44*  *C9orf129*  *C9orf3*  *CA5B*  *CACHD1*  *CACNA1D*  *CADM1*  *CALU*  *CAMTA1*  *CAPN7*  *CARD10*  *CASD1*  *CASP2*  *CAT*  *CBFB*  *CBLB*  *CBWD3*  *CBWD5*  *CCDC14*  *CCDC66*  *CCDC67*  *CCDC73*  *CCDC74A*  *CCL3L1*  *CCL3L3*  *CCL4L1*  *CCL4L2*  *CCND2*  *CCNI*  *CCNYL1*  *CCT8*  *CD109*  *CD2AP*  *CD81*  *CDC27*  *CDC42BPG*  *CDH11*  *CDH13*  *CDH4*  *CDK6*  *CDS2*  *CELF5*  *CENPI*  *CENPP*  *CEP112*  *CEP170*  *CEP41*  *CFI*  *CHD2*  *CHD6*  *CHD9*  *CHIC2*  *CHL1*  *CHSY3*  *CLDN11*  *CLEC2D*  *CLMP*  *CLN5*  *CMC1*  *CMIP*  *CMPK1*  *CMTM4*  *CMTM7*  *CNN3*  *CNNM3*  *CNTN1*  *CNTN4*  *CNTNAP2*  *COG3*  *COL16A1*  *COL1A1*  *COL22A1*  *COL27A1*  *COL3A1*  *COL4A1*  *COL5A2*  *COLEC12*  *COQ5*  *COTL1*  *CP*  *CPA6*  *CPEB3*  *CPNE1*  *CPSF6*  *CREB1*  *CREBBP*  *CRIM1*  *CRISPLD1*  *CRISPLD2*  *CRYBB2P1*  *CSAD*  *CSNK1A1*  *CSNK1G3*  *CSRNP3*  *CTDP1*  *CTDSPL*  *CTDSPL2*  *CTNNB1*  *CTNND1*  *CTR9*  *CTSK*  *CXADR*  *CXADRP3*  *CXorf23*  *CYP20A1*  *CYTH3*  *DACH1*  *DCLK1*  *DCLRE1C*  *DCTN6*  *DCUN1D1*  *DDR2*  *DDX39B*  *DEFB109P1*  *DEFB130*  *DHX36*  *DIRC3*  *DISC1*  *DLC1*  *DLEU1*  *DLEU2*  *DLG1*  *DLG2*  *DMD*  *DMP1*  *DMXL1*  *DNAH14*  *DNAH7*  *DNAJC3*  *DOCK1*  *DOCK7*  *DPYSL2*  *DPYSL3*  *DSN1*  *DTWD1*  *DVL2*  *DYNC1I1*  *DYNC1LI2*  *DYNLT1*  *E2F4*  *E2F5*  *EBAG9*  *EBF1*  *EBF2*  *ECM2*  *EFNA5*  *EFR3A*  *EGLN1*  *EGR1*  *EIF1AY*  *EIF2AK4*  *EIF2C1*  *EIF2S3*  *EIF3C*  *EIF3CL*  *EIF3L*  *EIF4B*  *EIF4E*  *EIF4G3*  *ELF2*  *ELK1*  *ELMO3*  *ELP2*  *EMCN*  *EML4*  *EMP2*  *ENAH*  *ENOX2*  *ENPP1*  *EPB41*  *EPB41L2*  *EPG5*  *EPHA4*  *EPHA7*  *EPHB2*  *ERC1*  *ERC2*  *ERVV-1*  *ERVV-2*  *ESPL1*  *ESYT2*  *ETF1*  *ETFA*  *ETNK1*  *ETV5*  *EVI2B*  *EVI5*  *EWSR1*  *EXOC2*  *EXOC3L1*  *EXOC4*  *EXOC6B*  *EXT2*  *EYA2*  *EZH2*  *FAF2*  *FAM101B*  *FAM102B*  *FAM105B*  *FAM111A*  *FAM114A1*  *FAM117A*  *FAM134B*  *FAM13A*  *FAM155A*  *FAM160B1*  *FAM168A*  *FAM172A*  *FAM178A*  *FAM204A*  *FAM208A*  *FAM27A*  *FAM49B*  *FAM60A*  *FAM66A*  *FAM82B*  *FAM86B1*  *FAT1*  *FAT4*  *FBN1*  *FBN2*  *FBXL17*  *FBXL20*  *FBXL7*  *FBXL8*  *FBXO42*  *FBXW7*  *FCHSD2*  *FER*  *FGF13*  *FHIT*  *FHOD1*  *FIGN*  *FKBP5*  *FLJ30838*  *FLJ39534*  *FLJ39739*  *FLJ45340*  *FLRT2*  *FLVCR1*  *FMNL2*  *FN1*  *FNBP1*  *FNBP1L*  *FNDC1*  *FNDC3A*  *FNDC3B*  *FOXD4L2*  *FOXD4L4*  *FOXD4L5*  *FOXN3*  *FOXO1*  *FOXO3*  *FOXP1*  *FOXP2*  *FREM1*  *FRMD4A*  *FRMD4B*  *FRMD8*  *FRS2*  *FRYL*  *FRZB*  *FSTL1*  *FSTL5*  *FTO*  *FTX*  *FUT8*  *FXR1*  *FYN*  *GABRG3*  *GALK2*  *GAS2L3*  *GAS7*  *GATAD2A*  *GBAS*  *GCFC2*  *GCLM*  *GCOM1*  *GFM2*  *GGNBP2*  *GHR*  *GIT2*  *GK5*  *GLB1*  *GLDN*  *GLT8D2*  *GLTSCR1*  *GLTSCR2*  *GMDS*  *GNA12*  *GNA13*  *GNAL*  *GNAQ*  *GNG12*  *GNG2*  *GNL3L*  *GOLGA3*  *GOLGA4*  *GOLGA6L10*  *GOLGA6L9*  *GPHN*  *GPI*  *GPKOW*  *GPM6B*  *GPR137B*  *GPR83*  *GRB2*  *GRIA3*  *GRIK2*  *GRIK3*  *GSK3B*  *GSN*  *GTDC1*  *GTF2H2*  *GTF2H2B*  *GTF2H2C*  *GTF2H2D*  *GTF2IP1*  *GUSBP3*  *GUSBP9*  *H3F3A*  *H3F3AP4*  *H3F3B*  *HADHB*  *HECA*  *HEG1*  *HEPH*  *HERC4*  *HEXB*  *HINT1*  *HLCS*  *HLTF*  *HMBOX1*  *HOMER1*  *HOMEZ*  *HOOK3*  *HP1BP3*  *HPN*  *HSF4*  *HSP90AA1*  *HSPA14*  *HSPA1A*  *HSPA1B*  *HSPA8*  *IBSP*  *ICA1L*  *IDE*  *IFIT5*  *IFT74*  *IFT80*  *IGF1R*  *IGF2BP2*  *IGFBP6*  *IGFBP7*  *IGLL3P*  *IGSF11*  *IL1RAPL1*  *IMPDH2*  *INE2*  *ING3*  *INTS4*  *INTS8*  *INTS9*  *INVS*  *IQCG*  *IQGAP2*  *IRF2*  *IRF2BP2*  *IRS1*  *ITFG1*  *ITGA8*  *ITGAV*  *ITGB7*  *ITGB8*  *ITPKC*  *ITPR2*  *ITSN1*  *JAK2*  *JDP2*  *JMJD1C*  *JPX*  *JUN*  *KALRN*  *KANSL3*  *KAT2B*  *KAT6B*  *KCND2*  *KCNQ1OT1*  *KCTD12*  *KCTD14*  *KCTD16*  *KCTD19*  *KDM4C*  *KDM5A*  *KIAA0247*  *KIAA0889*  *KIAA0895L*  *KIAA1109*  *KIAA1147*  *KIAA1199*  *KIAA1217*  *KIAA1244*  *KIAA1267*  *KIAA1432*  *KIAA1467*  *KIAA2026*  *KIF16B*  *KIF26B*  *KLHDC10*  *KLHL2*  *KLHL20*  *KLHL24*  *KLHL28*  *KLHL3*  *KPNA1*  *KPNA4*  *KRTAP1-1*  *KRTAP1-3*  *KRTAP2-1*  *KRTAP2-4*  *KRTAP4-11*  *KRTAP4-12*  *KRTAP4-4*  *KRTAP4-6*  *KRTAP4-7*  *KRTAP4-8*  *KRTAP4-9*  *KTN1*  *LAMA4*  *LAMB1*  *LAMB2*  *LARP4B*  *LCOR*  *LCORL*  *LDLRAD3*  *LEF1*  *LEFTY2*  *LGALSL*  *LGR5*  *LHFP*  *LIMA1*  *LIMCH1*  *LIN54*  *LIN7A*  *LIN7C*  *LINC00340*  *LINC00478*  *LMAN1*  *LOC100093631*  *LOC100128675*  *LOC100130000*  *LOC100132062*  *LOC100132287*  *LOC100132891*  *LOC100133267*  *LOC100133331*  *LOC100170939*  *LOC100272216*  *LOC100289255*  *LOC100379224*  *LOC100505678*  *LOC100505783*  *LOC100506071*  *LOC100506714*  *LOC100507217*  *LOC100507401*  *LOC100652768*  *LOC150568*  *LOC150776*  *LOC256021*  *LOC283585*  *LOC389043*  *LOC390660*  *LOC401010*  *LOC440297*  *LOC440300*  *LOC440910*  *LOC641518*  *LOC646719*  *LOC647859*  *LOC648740*  *LOC727849*  *LOC728407*  *LOC728558*  *LOC728855*  *LOC728875*  *LOC729177*  *LOC729737*  *LOC730755*  *LPAR1*  *LPHN2*  *LPHN3*  *LPP*  *LRBA*  *LRCH1*  *LRP1B*  *LRP4*  *LRP5L*  *LRRC16A*  *LRRC28*  *LRRC29*  *LRRC38*  *LRRC47*  *LRRC4C*  *LRRC8B*  *LRRFIP2*  *LRRTM4*  *LSAMP*  *LTBP1*  *LUC7L3*  *LYZ*  *MAGED1*  *MAGI1*  *MAGI3*  *MAP2K1*  *MAP3K12*  *MAP3K2*  *MAP4K5*  *MAP7D3*  *MAPK10*  *MAPK14*  *MAPKAPK5*  *MARCKS*  *MARK1*  *MARK3*  *MARK4*  *MBD2*  *MBD5*  *MBNL1*  *MBNL3*  *MBP*  *MDFIC*  *MDM4*  *MDN1*  *MEMO1*  *METTL21B*  *MEX3C*  *MFSD5*  *MGAT4C*  *MGC2752*  *MGEA5*  *MIA3*  *MID1*  *MIER1*  *MINPP1*  *MIR100HG*  *MIR1279*  *MIR181A2HG*  *MIR198*  *MIR3134*  *MIR3180-4*  *MIR328*  *MIR4784*  *MIR5095*  *MIR548AM*  *MIR548F5*  *MIR548H4*  *MIR548N*  *MIR548W*  *MKX*  *MLIP*  *MLL*  *MLL3*  *MLL5*  *MLLT10*  *MLLT3*  *MMP13*  *MMP9*  *MMS19*  *MMS22L*  *MORF4L1*  *MPHOSPH8*  *MRPL3*  *MRPS6*  *MSL2*  *MSMO1*  *MSRA*  *MTERFD1*  *MTF2*  *MTHFD2*  *MTR*  *MUC21*  *MUC22*  *MYNN*  *MYPOP*  *MZT2A*  *N4BP2L2*  *NAA16*  *NAIP*  *NAP1L1*  *NARG2*  *NAT10*  *NBPF10*  *NBPF11*  *NBPF14*  *NBPF15*  *NBPF16*  *NBPF24*  *NCAM1*  *NCBP1*  *NCKAP5*  *NCOA1*  *NDEL1*  *NDFIP1*  *NDUFB8*  *NDUFC2-KCTD14*  *NDUFS2*  *NDUFV2*  *NEFL*  *NEK1*  *NELL1*  *NES*  *NF1*  *NFIA*  *NFIB*  *NFIX*  *NFX1*  *NHLRC2*  *NHS*  *NID2*  *NKAIN2*  *NKTR*  *NLGN1*  *NLRP4*  *NMNAT2*  *NNAT*  *NOL3*  *NOLC1*  *NONO*  *NOTCH2*  *NPAS3*  *NPHP3*  *NPHP3-ACAD11*  *NPLOC4*  *NPNT*  *NR2C2*  *NR3C2*  *NR6A1*  *NREP*  *NRP2*  *NRXN1*  *NRXN3*  *NSD1*  *NT5C2*  *NTM*  *NTRK2*  *NTRK3*  *NUMA1*  *NUP214*  *NUP93*  *NUSAP1*  *OCLN*  *OCRL*  *ODF3*  *ODZ2*  *ODZ3*  *ODZ4*  *OGN*  *OGT*  *OLA1*  *OLFML1*  *OLFML2A*  *OPHN1*  *OPN1SW*  *OR4F16*  *OR4F29*  *OR4F3*  *ORC4*  *OSBPL11*  *OSBPL3*  *OSTF1*  *OTOR*  *OTUD7A*  *P4HA1*  *PAFAH1B2*  *PAN3*  *PAPD4*  *PARD3*  *PARD3B*  *PARG*  *PARP14*  *PBRM1*  *PBX1*  *PCBP1-AS1*  *PCBP2*  *PCDH18*  *PCDH9*  *PCDHB11*  *PCF11*  *PCM1*  *PCSK7*  *PCYOX1*  *PCYT1B*  *PDCD4*  *PDE4B*  *PDE4D*  *PDE5A*  *PDE7B*  *PDGFC*  *PDGFD*  *PDGFRA*  *PDS5B*  *PDZD2*  *PDZRN3*  *PEX5L*  *PFDN5*  *PGAP1*  *PGGT1B*  *PHACTR4*  *PHF12*  *PHIP*  *PHKG2*  *PHLPP2*  *PHTF1*  *PI15*  *PI4KA*  *PIAS1*  *PIAS2*  *PICALM*  *PIGF*  *PIK3R4*  *PIP4K2A*  *PKD2*  *PKIG*  *PLCB1*  *PLCB4*  *PLCE1*  *PLCL2*  *PLEC*  *PLEKHG4*  *PLXDC2*  *POGZ*  *POLL*  *POLN*  *POLR2M*  *POTEKP*  *POU2F1*  *PPAPDC1A*  *PPFIBP1*  *PPIAL4A*  *PPIAL4B*  *PPIAL4C*  *PPIAL4D*  *PPIAL4E*  *PPIAL4F*  *PPL*  *PPM1H*  *PPP1R12A*  *PPP2R2B*  *PPP2R3A*  *PPP2R5E*  *PPP3CA*  *PPP6R2*  *PPWD1*  *PRDM2*  *PRG4*  *PRH1-PRR4*  *PRICKLE2*  *PRKAG2*  *PRKCA*  *PRKCI*  *PRKD1*  *PRKD3*  *PRKG1*  *PRKRA*  *PRMT2*  *PRND*  *PRR13*  *PRR14L*  *PRRX1*  *PSD3*  *PSEN1*  *PTAR1*  *PTBP2*  *PTCH1*  *PTEN*  *PTGIS*  *PTK2*  *PTN*  *PTP4A2*  *PTPDC1*  *PTPN12*  *PTPN14*  *PTPN4*  *PTPRA*  *PTPRB*  *PTPRG*  *PTPRJ*  *PTPRK*  *PTPRM*  *PWWP2A*  *PZP*  *QKI*  *QSOX1*  *RAB10*  *RAB12*  *RAB1A*  *RAB21*  *RAB2A*  *RAB31*  *RAB3GAP1*  *RAB3GAP2*  *RAB4A*  *RAB5A*  *RAB6A*  *RABGAP1*  *RABGAP1L*  *RAD51B*  *RAD51L3-RFFL*  *RAI14*  *RANBP3*  *RANBP3L*  *RANBP9*  *RAP1A*  *RAP1B*  *RAP2A*  *RAP2B*  *RAPGEF2*  *RAPGEF5*  *RARG*  *RARS2*  *RASAL2*  *RASGRF2*  *RASSF8*  *RB1CC1*  *RBFOX1*  *RBL1*  *RBM26*  *RBM6*  *RBMS3*  *RBPJ*  *RCOR3*  *REEP3*  *RELL1*  *RELN*  *RFC3*  *RFFL*  *RGL1*  *RGS10*  *RGS12*  *RGS6*  *RHD*  *RHOBTB1*  *RICTOR*  *RIT1*  *RLF*  *RNF111*  *RNF115*  *RNF144A*  *RNF144B*  *RNF145*  *RNF214*  *RNF31*  *ROCK2*  *RORA*  *RPH3AL*  *RPL23*  *RPL35A*  *RPL4*  *RPRD2*  *RPS17*  *RPS17L*  *RPS19*  *RSF1*  *RSU1*  *RUNDC3B*  *RUNX1*  *SAE1*  *SAMD12*  *SAMHD1*  *SAR1A*  *SASH1*  *SAT1*  *SATB2*  *SAYSD1*  *SBF2*  *SBNO1*  *SCAF11*  *SCAF8*  *SCMH1*  *SCN7A*  *SCYL3*  *SDK1*  *SEC11C*  *SEC16B*  *SEC23IP*  *SEC24D*  *SEC61A1*  *SECISBP2L*  *SEMA3B*  *SEMA3D*  *SEMA3E*  *SEMA5A*  *SEMA6A*  *SENP6*  *SENP7*  *SEPHS1*  *SEPT2*  *SEPT7*  *SERF1A*  *SERF1B*  *SERINC5*  *SERPINF1*  *SERPINH1*  *SESN3*  *SESTD1*  *SETBP1*  *SFXN1*  *SGCD*  *SGPL1*  *SH3BP5*  *SH3D19*  *SH3PXD2B*  *SH3TC2*  *SHC4*  *SHISA2*  *SIDT2*  *SIK2*  *SIK3*  *SIKE1*  *SIRT1*  *SKA2*  *SLC14A2*  *SLC16A10*  *SLC20A2*  *SLC22A23*  *SLC25A32*  *SLC25A37*  *SLC26A2*  *SLC35F1*  *SLC35F2*  *SLC39A10*  *SLC7A2*  *SLC9A5*  *SLIT2*  *SLIT3*  *SMA4*  *SMA5*  *SMAD2*  *SMAD3*  *SMARCA5*  *SMARCB1*  *SMARCC1*  *SMN1*  *SMN2*  *SMOC2*  *SMYD3*  *SMYD4*  *SNCA*  *SNORD18C*  *SNTB2*  *SNX14*  *SNX19*  *SNX24*  *SOAT2*  *SOBP*  *SORBS2*  *SOS1*  *SOX2-OT*  *SOX4*  *SOX5*  *SOX6*  *SOX9*  *SP1*  *SP7*  *SPAG16*  *SPAG9*  *SPARC*  *SPATA6*  *SPATS2*  *SPECC1*  *SPHAR*  *SPP1*  *SPPL3*  *SPRY2*  *SPTBN1*  *SRBD1*  *SREBF2*  *SRGAP2*  *SRGAP2P2*  *SRGN*  *SRPRB*  *SRSF3*  *SRSF5*  *SS18*  *SSBP2*  *SSH2*  *SSR1*  *SSR2*  *ST13*  *ST3GAL3*  *ST3GAL6*  *ST6GAL2*  *ST6GALNAC2*  *ST8SIA1*  *STAC*  *STAG1*  *STAG2*  *STARD7*  *STAT1*  *STK39*  *STON2*  *STRN3*  *STT3A*  *STX2*  *STXBP6*  *SUDS3*  *SUFU*  *SUPT3H*  *SUSD1*  *SUV420H1*  *SYCP1*  *SYN3*  *SYT1*  *TAGLN*  *TAMM41*  *TANC1*  *TANC2*  *TANK*  *TAOK1*  *TAOK3*  *TASP1*  *TBC1D20*  *TBC1D23*  *TBC1D3B*  *TBCA*  *TBL1XR1*  *TBX18*  *TCF4*  *TCFL5*  *TDRD3*  *TEAD1*  *TFPI*  *TGFBI*  *TGFBR3*  *THAP5*  *THBS2*  *THSD4*  *THSD7B*  *TIMP3*  *TJP1*  *TLE4*  *TM9SF2*  *TM9SF3*  *TMCO7*  *TMEM117*  *TMEM131*  *TMEM159*  *TMEM161B*  *TMEM208*  *TMEM87B*  *TMOD3*  *TMTC3*  *TMX2-CTNND1*  *TNFRSF10B*  *TNKS*  *TNKS2*  *TNPO1*  *TNPO3*  *TNRC6A*  *TNRC6B*  *TNS3*  *TNXB*  *TOP1*  *TOPORS*  *TPR*  *TPT1-AS1*  *TRADD*  *TRAF3IP1*  *TRAF3IP2-AS1*  *TRAPPC9*  *TRDMT1*  *TRIL*  *TRIM33*  *TRIM9*  *TRIP12*  *TRMT61B*  *TRPM3*  *TRPS1*  *TRPV2*  *TSC22D1*  *TSC22D2*  *TSHR*  *TSIX*  *TSNAX-DISC1*  *TSPAN3*  *TSPAN7*  *TSPYL1*  *TTC1*  *TTC28*  *TTC3*  *TTL*  *TTLL5*  *TUBA3D*  *TUBGCP4*  *TUG1*  *TULP3*  *TWF1*  *U2SURP*  *UACA*  *UBC*  *UBE2B*  *UBE2D3*  *UBE2E1*  *UBE2E3*  *UBE2H*  *UBE2N*  *UBE2Q2P2*  *UBE2Q2P3*  *UBE2W*  *UBR2*  *UGGT2*  *UGT8*  *UHRF2*  *ULK4*  *UNC5C*  *UNC5D*  *UPF2*  *UQCC*  *USP13*  *USP34*  *USP54*  *USP6NL*  *UST*  *UTRN*  *UVRAG*  *VANGL1*  *VASH2*  *VCAN*  *VCL*  *VEZT*  *VIM*  *VPS45*  *VPS54*  *WAC*  *WARS*  *WBSCR17*  *WDFY3*  *WDHD1*  *WDPCP*  *WDR26*  *WDR52*  *WDR59*  *WDR60*  *WDR81*  *WNT5A*  *WRN*  *WTH3DI*  *WTIP*  *WWC2*  *WWOX*  *WWTR1*  *XIST*  *XYLT1*  *YEATS2*  *YEATS4*  *YES1*  *YIPF6*  *YLPM1*  *YWHAQ*  *YWHAZ*  *ZBTB1*  *ZBTB16*  *ZBTB20*  *ZBTB44*  *ZC3H11A*  *ZC3H13*  *ZC3H6*  *ZCCHC16*  *ZEB1*  *ZEB2*  *ZFAND3*  *ZFHX3*  *ZFHX4*  *ZFP90*  *ZFYVE20*  *ZIC2*  *ZIC5*  *ZMYM2*  *ZMYM4*  *ZMYND11*  *ZNF137P*  *ZNF160*  *ZNF233*  *ZNF236*  *ZNF259*  *ZNF28*  *ZNF280C*  *ZNF281*  *ZNF286A*  *ZNF300*  *ZNF300P1*  *ZNF320*  *ZNF321P*  *ZNF347*  *ZNF37A*  *ZNF37BP*  *ZNF407*  *ZNF415*  *ZNF429*  *ZNF451*  *ZNF468*  *ZNF503*  *ZNF503-AS2*  *ZNF516*  *ZNF518B*  *ZNF547*  *ZNF559-ZNF177*  *ZNF562*  *ZNF578*  *ZNF600*  *ZNF608*  *ZNF611*  *ZNF628*  *ZNF638*  *ZNF665*  *ZNF675*  *ZNF701*  *ZNF702P*  *ZNF704*  *ZNF710*  *ZNF711*  *ZNF740*  *ZNF808*  *ZNF812*  *ZNF816*  *ZNF816-ZNF321P*  *ZNF827*  *ZNF83*  *ZSWIM6*  *ZWINT* | *ADAMTS1*  *ADAMTS20*  *ALG10B*  *ANKRD6*  *ARHGAP12*  *ATG5*  *ATP10B*  *ATP11A*  *ATP2A3*  *BMI1*  *BMP7*  *CAB39L*  *CADM2*  *CALB1*  *CAMPSAP3*  *CDH13*  *CEP104*  *CTIF*  *CTNNA1*  *CXADR*  *DBN1*  *DCLK1*  *DCT*  *DDR1*  *DSG1*  *ELP3*  *EMX2*  *EPHA1*  *ESRRG*  *ESSRG*  *EZH2*  *FAM173B*  *FAM43A*  *FBXO30*  *FOXI2*  *FOXO3*  *FRMPD4*  *FSCN2*  *GFI1*  *GJB4*  *GRHL1*  *HAS1*  *HCN1*  *HERTC3*  *HMGA2*  *HOXB1*  *HOXB2*  *HPN*  *HYAL2*  *IKZF2*  *KCNA10*  *KCNK5*  *KCNRG*  *KIAA1731*  *KLHDC7B*  *KLHL18*  *KPTN*  *LAMC1*  *LMO4*  *LRRC4C*  *MAP3K1*  *MCOLN2*  *MCOLN3*  *MEPE*  *MIR124-2*  *MIR15A*  *MIR182*  *MIR183*  *MIR18A*  *MIR204*  *MRPS2*  *MVD*  *MX1*  *MYH13*  *MYH7B*  *MYO1F*  *NEDD4*  *NEFH*  *NOG*  *NOXO1*  *NPTN*  *OCLN*  *OCM*  *ODF2*  *PA2G4*  *PANX1*  *PCDH1*  *PCDH20*  *PCDHGA10*  *PDXDC1*  *PIEZO1*  *PKHD1L1*  *PLXNA3*  *PTX3*  *PXN*  *RIG1*  *SCARB2*  *SCNN1A*  *SCNN1B*  *SEMA4A*  *SERPINF1*  *SESN2*  *SFSWAP*  *SIK3*  *SIRT1*  *SLC15A5*  *SLC28A3*  *SLC3A1*  *SLC44A2*  *SLC4A2*  *SLC4A9*  *SLC7A8*  *SLC9A3R2*  *SORCS2*  *SPATC1L*  *SRRM4*  *SSBP1*  *STK11*  *STRN*  *SVIL*  *SYNJ2*  *TAOK1*  *TBCE*  *TECTB*  *THRAP3*  *TMCC2*  *TOGARAM2*  *TUBB4B*  *TUSC2*  *TYRP1*  *USP31*  *USP53*  *WRB*  *XIRP2*  *YWHAE*  *ZBTB16*  *ZIC2* |

** Genes that are associated with both non-syndromic and syndromic deafness in humans. ^#^* *Subgroup of mouse deafness genes that was used in the analysis of group AD based on personal communication and literature, no genes were added to this list after August 4 2021.*
