# Supplementary material for: Exome variant prioritization in a large cohort of hearing-impaired individuals indicates IKZF2 to be associated with non-syndromic hearing loss and guides future research of unsolved cases
Source: Hum Genet. 2024 Oct 16;143(11):1379–99. doi: 10.1007/s00439-024-02706-w (PMC11522133; doi:10.1007/s00439-024-02706-w)
Supplement: Supplementary file 5 — Supplementary file5 (DOCX 13 KB) [file 439_2024_2706_MOESM5_ESM.docx]

**Supplemental Table 2. Flowchart of variant filtering in group AR, known human deafness genes.**

| 900,417 variants | Selection: human deafness genes  Excluded: 898,166 variants |
| --- | --- |
| 2,247 variants | Selection: ≥2 variants per sample or homozygous  Excluded: 1,932 variants |
| 315 variants | Selection: ≥1 truncating variant  Excluded: 213 variants |
| 102 variants | Selection: excluding artefacts* (alignment files)  Excluded: 92 variants |
| 10 variants | Selection: clinical files; literature  Excluded: 5 variants (Supplemental Table 3) |
| Follow-up: 5 variants (Table 1) | |

List 1: human deafness genes. * And <2 variants per sample and not homozygous.
