# Supplementary material for: Exome variant prioritization in a large cohort of hearing-impaired individuals indicates IKZF2 to be associated with non-syndromic hearing loss and guides future research of unsolved cases
Source: Hum Genet. 2024 Oct 16;143(11):1379–99. doi: 10.1007/s00439-024-02706-w (PMC11522133; doi:10.1007/s00439-024-02706-w)
Supplement: Supplementary file 6 — Supplementary file6 (DOCX 16 KB) [file 439_2024_2706_MOESM6_ESM.docx]

**Supplemental Table 3. Excluded variants in group AR.**

| **Gene** | **Variant** | **Reason for exclusion**^#^ |
| --- | --- | --- |
| **Human deafness genes (list 1)** | | |
| *ALG11* | Chr13(GRCh37):g.52593105G>A  NM_001004127.3:c.101G>A  p.(Cys34Tyr) | Different from phenotype reported in the literature |
|  | Chr13(GRCh37):g.52602723_52602726del  NM_001004127.3:c.1476_1479del  p.(Lys492Asnfs*36) |  |
| *ALMS1* | Chr2(GRCh37):g.73613168T>C  NM_015120.2:c.172T>C  p.(Ser58Pro) | Different from phenotype reported in the literature |
|  | Chr2(GRCh37):g.73800386del  NM_015120.2:c.11379del  p.(Phe3793Leufs*38) |  |
| *GAS2* | Chr11(GRCh37):g.22777500G>A  NM_001391933.1:c.723+1G>A  p.? (homozygous) | Case had already been solved with this variant but was not correctly removed from the cohort |
| **Candidate deafness genes (lists 2, 3, 4)** | | |
| *ABCA8* | Chr17(GRCh37):g.66871544G>A  NM_007168.4:c.4371-3C>T  p.? | Case was solved after inclusion |
|  | Chr17(GRCh37):g.66890307T>C  NM_007168.4:c.2916+7A>G  p.? |  |
| *ADAMTS20* | Chr12(GRCh37):g.43822279T>C  NM_025003.5:c.3710A>G  p.(Tyr1237Cys) | Case was solved after inclusion |
|  | Chr12(GRCh37):g.43822597G>A  NM_025003.5:c.3495C>T  p.(Ser1165=) |  |
| *CSE1L* | Chr20(GRCh37):g.47693593C>T  NM_001316.4:c.1412C>T  p.(Ser471Leu) | Subject withdrawn |
| *NBEAL1* | Chr2(GRCh37):g.204013762A>T  NM_001114132.2:c.5266A>T  p.(Arg1756Trp) | Subject withdrawn |
|  | Chr2(GRCh37):g.204073957C>T  NM_001114132.2:c.7610C>T  p.(Ser2537Phe) |  |
| *NRP1* | Chr10(GRCh37):g.33510772T>C  NM_003873.7:c.1157A>G  p.(Asn386Ser) | Likely other cause (post-meningitis) |
|  | Chr10(GRCh37):g.33515199G>A  NM_003873.7:c.1000C>T  p.(Arg334Cys) |  |
| *PLXNA3* | ChrX(GRCh37):g.153690368G>A  NM_017514.5:c.1135-100G>A  p.? (hemizygous) | Likely environmental cause (late-onset) |
| *RGN* | ChrX(GRCh37):g.46951526A>C  NM_152869.4):c.761A>C  p.(Asn254Thr) (hemizygous) | Likely environmental cause (late-onset) |
| *RNF144A* | Chr2(GRCh37):g.7137040G>A  NM_014746.6:c.-11-7G>A  p.? (homozygous) | Subject withdrawn |
| *TOGARAM2* | Chr2(GRCh37):g.29240725del  NM_199280.4:c.1263del  p.(Asn421Lysfs*7) | Case was solved after inclusion |
|  | Chr2(GRCh37):g.29247243A>G  NM_199280.4:c.1853+3A>G  p.? |  |
| *TRPM3* | Chr9(GRCh37):g.73235164_73235177del  NM_001366145.2:c.1944_1957del  p.(His649Valfs*19) | Likely environmental cause (late-onset) |
|  | Chr9(GRCh37):g.73391371C>T  ENST00000396283:c.797G>A  p.(Arg266Lys) |  |

Variants are in heterozygous state unless stated otherwise. ^#^ One cell per subject.
