# Supplementary material for: Exome variant prioritization in a large cohort of hearing-impaired individuals indicates IKZF2 to be associated with non-syndromic hearing loss and guides future research of unsolved cases
Source: Hum Genet. 2024 Oct 16;143(11):1379–99. doi: 10.1007/s00439-024-02706-w (PMC11522133; doi:10.1007/s00439-024-02706-w)
Supplement: Supplementary file 7 — Supplementary file7 (DOCX 13 KB) [file 439_2024_2706_MOESM7_ESM.docx]

**Supplemental Table 4. Flowchart of variant filtering in group AR, candidate deafness genes.**

| 900,417 variants | Selection: candidate deafness genes  Excluded: 887,384 variants |
| --- | --- |
| 13,033 variants | Selection: ≥2 variants per sample or homozygous  Excluded: 10,805 variants |
| 2,228 variants | Selection: genes not linked to OMIM phenotypes  Excluded: 905 variants |
| 1,323 variants | Selection: *in silico* prediction scores*  Excluded: 735 variants |
| 588 variants | Selection: excluding artefacts^#^ (alignment files)  Excluded: 546 variants |
| 42 variants | Selection: clinical files; literature  Excluded: 16 variants (Supplemental Table 3) |
| Follow-up: 26 variants (Table 2, Supplemental Table 5) | |

List 2: mouse deafness genes; list 3: genes with preferential inner ear expression; list 4: other candidate genes. * And ≥2 variants per sample or homozygous; ^#^ And <2 variants per sample and not homozygous.
