# Supplementary material for: Exome variant prioritization in a large cohort of hearing-impaired individuals indicates IKZF2 to be associated with non-syndromic hearing loss and guides future research of unsolved cases
Source: Hum Genet. 2024 Oct 16;143(11):1379–99. doi: 10.1007/s00439-024-02706-w (PMC11522133; doi:10.1007/s00439-024-02706-w)
Supplement: Supplementary file 8 — Supplementary file8 (DOCX 15 KB) [file 439_2024_2706_MOESM8_ESM.docx]

**Supplemental Table 5. Excluded variants in follow-up of candidate deafness genes in group AR.**

| **Gene** | **Variant** | **Findings^#^** |
| --- | --- | --- |
| *CHD6* | Chr20(GRCh37):g.40120435G>T  NM_032221.5:c.1339C>A  p.(Gln447Lys) | Variants do not co-segregate with HL |
|  | Chr20(GRCh37):g.40117215del  NM_032221.5:c.1711del  p.(His571Thrfs*9) |  |
| *FAT1* | Chr4(GRCh37):g.187629951G>A  NM_005245.4:c.1031C>T  p.(Pro344Leu) | Variants do not co-segregate with HL |
|  | Chr4(GRCh37):g.187540380G>A  NM_005245.4:c.7360C>T  p.(Arg2454Trp) |  |
| *GRHL1* | Chr2(GRCh37):g.10104077C>T NM_198182.3:c.809C>T  p.(Thr270Ile) | Variants do not co-segregate with HL |
|  | Chr2(GRCh37):g.10101437G>T NM_198182.3:c.541G>T  p.(Glu181*) |  |
| *LRP1B* | Chr2(GRCh37):g.141143522C>T  NM_018557.3:c.10471G>A  p.(Asp3491Asn) | Variants do not co-segregate with HL |
|  | Chr2(GRCh37):g.141747181C>T  NM_018557.3:c.2690G>A  p.(Arg897His) |  |
| *MAGI3* | Chr1(GRCh37):g.114214427C>T  NM_001142782.2:c.2887C>T  p.(Gln963*) | Variants do not co-segregate with HL |
|  | Chr1(GRCh37):g.114226506_114226507del  NM_001142782.2:c.4316_4317del  p.(Lys1439Argfs*14) |  |
| *MAP7D3* | ChrX(GRCh37):g.135313127T>C  NM_024597.4:c.1414-2A>G  p.? (hemizygous) | Variant could not be validated by PCR and Sanger sequencing |
| *MYH13* | Chr17(GRCh37):g.10215936G>T  NM_003802.3:c.4320C>A  p.(His1440Gln) | Variant c.2411A>T could not be validated by PCR and Sanger sequencing |
|  | Chr17(GRCh37):g.10233728T>A  NM_003802.3:c.2411A>T  p.(Glu804Val) |  |
|  | Chr17(GRCh37):g.10222198C>T  NM_003802.3:c.3647G>A  p.(Arg1216Gln) | PCR and Sanger sequencing showed variants to be *in cis* |
|  | Chr17(GRCh37):g.10227550C>T  NM_003802.3:c.2723G>A  p.(Arg908Gln) |  |
| *RGN* | ChrX(GRCh37): g.46940340G>A  NM_152869.4:c.-16+5G>A  p.? (hemizygous) | Variant could not be validated by PCR and Sanger sequencing |
|  | Chr6(GRCh37):g.146271582T>G NM_001042683.1:c.800A>C  p.(Glu267Ala) |  |
| *SIKE1* | Chr1(GRCh37):g.115319080_115319081ins44  NM_001102396.1:c.421-1_421ins44  p.? | Variants could not be validated by PCR and Sanger sequencing |
|  | Chr1(GRCh37):g.115323203_115323204ins42  NM_001102396.1: c.26_27ins42  p.(Thr10_Lys211delinsGluTyrIlePheCysThr) |  |

Variants are in heterozygous state unless stated otherwise. ^#^ One cell per subject.
