# Supplementary material for: Exome variant prioritization in a large cohort of hearing-impaired individuals indicates IKZF2 to be associated with non-syndromic hearing loss and guides future research of unsolved cases
Source: Hum Genet. 2024 Oct 16;143(11):1379–99. doi: 10.1007/s00439-024-02706-w (PMC11522133; doi:10.1007/s00439-024-02706-w)
Supplement: Supplementary file 9 — Supplementary file9 (DOCX 13 KB) [file 439_2024_2706_MOESM9_ESM.docx]

**Supplemental Table 6. Flowchart of variant filtering in group AR, copy number variants in known human deafness genes (H) and candidate deafness genes (C).**

| 900,417 variants | Selection: monoallelic truncating variants in human deafness genes (list 1) or candidate deafness genes (lists 2, 3, 4)  Excluded: 899,755 variants |
| --- | --- |
| 81 (H) + 581 (C) variants | Selection: CNV file available  Excluded: 309 variants |
| 43 (H) + 310 (C) variants | Selection: CNV called in the same gene  Excluded: 339 variants |
| 2 (H) + 12 (C) variants | Selection: highly variable genes excluded  Excluded: 6 variants |
| Follow-up: 2 (H) + 6 (C) variants (Supplemental Table 6) | |

CNV, copy number variation.
