# Supplementary material for: Exome variant prioritization in a large cohort of hearing-impaired individuals indicates IKZF2 to be associated with non-syndromic hearing loss and guides future research of unsolved cases
Source: Hum Genet. 2024 Oct 16;143(11):1379–99. doi: 10.1007/s00439-024-02706-w (PMC11522133; doi:10.1007/s00439-024-02706-w)
Supplement: Supplementary file 10 — Supplementary file10 (DOCX 15 KB) [file 439_2024_2706_MOESM10_ESM.docx]

**Supplemental Table 7. Copy number variants in known human deafness genes and candidate deafness genes.**

| **Gene** | **Variant** | **Remark** | **Result qPCR analysis** |
| --- | --- | --- | --- |
| *AGBL3* | Chr7(GRCh37):g.134672501_134674220del  NM_178563.4:c.-59-125_124+159del  p.Met1? |  | Not confirmed |
| *EXD2* | Chr14(GRCh37):g.69707461_69727245dup  NM_001193360.2:c.1650-140_*19428dup  p.? |  | Not confirmed |
| *MGAM* | Chr7(GRCh37):g.141760036_141760375del  NM_001365693.1:c.4060-75_4122+202del  p.(Val1354_Glu1374del) | Overlapping calls, representing one single CNV | CNV confirmed, segregation analysis showed the CNV to be *in cis* with the truncating variant |
|  | Chr7(GRCh37):g.141765450_141786135del  NM_001365693.1:c.4653+28_6747+7del  p.(Thr1552_Lys2249del) |  |  |
|  | Chr7(GRCh37):g.141791631_141794497del  NM_001365693.1:c.6811-82_7341+43del  p.(Leu2271_Tyr2447del) |  |  |
|  | Chr7(GRCh37):g.141765620_141794327del  NM_001365693.1:c.4759_7306+30del  p.(Ile1587Alafs*2) | Potentially the same CNV as the other MGAM CNV | The same truncating and potentially the same CNV as the case above, thus presumed to be *in cis* as well |
| *NAT10* | Chr11(GRCh37):g.34167617_34167856del  NM_024662.3:c.2970-14_*117del  p.(Asp991Glufs*17) |  | Not confirmed |
| *POSTN* | Chr13(GRCh37):g.38148581_38153574del  NM_006475.3:c.1661-77_2089+129del  p.? |  | Not confirmed |
| *REST* | Chr4(GRCh37):g.57796490_57796829du  NM_005612.5:c.1466_1805dup  p.(Glu602Aspfs*27) |  | Not confirmed |
| *COL11A1* | Chr1(GRCh37):g.103379768_103380525del  NM_001854.4:c.3817-158_3978+140del  p.? | Both variants in one sample | Not confirmed |
|  | Chr1(GRCh37):g.103444962_103455268del  NM_001854.4:c.2341-140_2587del  p.(Gly853Profs*10) |  | Not confirmed |
