# Supplementary material for: Exome variant prioritization in a large cohort of hearing-impaired individuals indicates IKZF2 to be associated with non-syndromic hearing loss and guides future research of unsolved cases
Source: Hum Genet. 2024 Oct 16;143(11):1379–99. doi: 10.1007/s00439-024-02706-w (PMC11522133; doi:10.1007/s00439-024-02706-w)
Supplement: Supplementary file 11 — Supplementary file11 (DOCX 13 KB) [file 439_2024_2706_MOESM11_ESM.docx]

**Supplemental Table 8. Flowchart of variant filtering in group AD, known human deafness genes.**

| 27,089 variants | Selection: human deafness genes  Excluded: 26,998 variants |
| --- | --- |
| 91 variants | Selection: *in silico* prediction scores  Excluded: 20 variants |
| 71 variants | Selection: records clinical exome sequencing  Excluded: 18 variants |
| 53 variants | Selection: excluding artefacts (alignment files)  Excluded: 17 variants |
| 36 variants | Selection: MutationTaster update; clinical files; literature  Excluded: 29 variants (Supplemental Table 8) |
| Follow-up: 7 variants (Supplemental Table 10) | |

List 1: human deafness genes.
