# Supplementary material for: Exome variant prioritization in a large cohort of hearing-impaired individuals indicates IKZF2 to be associated with non-syndromic hearing loss and guides future research of unsolved cases
Source: Hum Genet. 2024 Oct 16;143(11):1379–99. doi: 10.1007/s00439-024-02706-w (PMC11522133; doi:10.1007/s00439-024-02706-w)
Supplement: Supplementary file 12 — Supplementary file12 (DOCX 16 KB) [file 439_2024_2706_MOESM12_ESM.docx]

**Supplemental Table 9. Excluded variants in human deafness genes in group AD.**

| **Gene** | **Variant** | **Reason for exclusion**^#^ |
| --- | --- | --- |
| *ABCC1* | Chr16(GRCh37):g.16173295T>C  ENST00000399408.6_2:c.1097T>G  p.(Leu366Trp) | Different from phenotype reported in the literature |
| *COL11A1* | Chr21(GRCh37):g.103496780C>A  NM_001854.4:c.672G>T  p.(Leu224Phe) | Different from phenotype reported in the literature |
|  | Chr21(GRCh37):g.103573638C>T  NM_001190709.2:c.97G>A  p.(Val33Ile) | *In silico* prediction tools (MutationTaster update) |
| *COL11A2* | Chr6(GRCh37):g.33141305C>T  NM_080679.3:c.2335G>A  p.(Gly779Arg) | Suspected inheritance pattern was not autosomal dominant |
| *DMXL2* | Chr15(GRCh37):g.51757783A>C  NM_001378458.1:c.7583T>G  p.(Ile2528Ser) | Different from phenotype reported in the literature |
|  | Chr15(GRCh37):g.51830610T>G  NM_001378458.1:c.1145A>C  p.(Asp382Ala) | Different from phenotype reported in the literature |
|  | Chr15(GRCh37):g.51778389C>G  NM_001378458.1:c.5363G>C  p.(Cys1788Ser) | No phenotypic information available, subject not accessible |
| *GATA3* | Chr10(GRCh37):g.8100313G>T  NM_001002295.2(GATA3):c.287G>T  p.(Trp96Leu) | Different from phenotype reported in the literature |
|  | Chr10(GRCh37): g.8097827C>A  NM_001002295.2(GATA3):c.209C>A  p.(Ala70Asp) | Different from phenotype reported in the literature |
| *GREB1L* | Chr18(GRCh37):g.19080184G>A  ENST00000269218.10_1.1:c.3559G>A  p.(Ala1187Thr) | Different from phenotype reported in the literature |
| *GSDME* | Chr7(GRCh37)g.24758753_24758755del  NM_001127453.2:c.491_493del  p.(Thr164del) | Different from specific variants reported in the literature |
| *MYH9* | Chr22(GRCh37):g.36678739C>A  NM_002473.6:c.5858G>T  p.(Gly1953Val) | Different from specific variants reported in the literature and *in silico* prediction tools (MutationTaster update) |
|  | Chr22(GRCh37):g.36689533C>T  NM_002473.6:c.3943-6G>A  p.? | Different from specific variants reported in the literature |
|  | Chr22(GRCh37):g.36697053C>A  NM_002473.6:c.2682G>T  p.(Glu894Asp) | Different from specific variants reported in the literature and *in silico* prediction tools (MutationTaster update) |
| *MYO3A* | Chr10(GRCh37):g.26446155A>T  ENST00000543632.5_2.1:c.1777-54617A>T  p.? | Different from specific variants reported in the literature |
| *PI4KB* | Chr1(GRCh37):g.151266848A>C  NM_001369626.1:c.2148+37T>G  p.? | Different from specific variants reported in the literature |
| *REST* | Chr4(GRCh37):g.57777681C>A  NM_005612.5:c.877C>A  p.(Gln293Lys) | Different from specific variants reported in the literature |
| *RIPOR2* | Chr6(GRCh37):g.24865635T>C  NM_001286447.2:c.458A>G  p.(Gln153Arg) | Suspected inheritance pattern was not autosomal dominant |
| *TBC1D24* | Chr16(GRCh37):g.2550870G>A  NM_020705.3:c.1573G>A  p.(Asp525Asn) | *In silico* prediction tools (MutationTaster update) |
| *TMC1* | Chr9(GRCh37):g.75445360A>T  NM_138691.3:c.2130-7A>T  p.? | Different from specific variants reported in the literature |
|  | Chr9(GRCh37):g.75445361A>T  NM_138691.3:c.2130-6A>T  p.? | Different from specific variants reported in the literature |
|  | Chr9(GRCh37):g.75369753G>A  NM_138691.3:c.694G>A  p.(Ala232Thr) | Suspected inheritance pattern was not autosomal dominant |
|  | Chr9(GRCh37):g.75450968C>A  NM_138691.3:c.*79C>A  p.? | Suspected inheritance pattern was not autosomal dominant |
| *TNC* | Chr9(GRCh37):g.117808955T>C  ENST00000341037.8_3.1:c.4313A>G  p.(Glu1438Gly) | Suspected inheritance pattern was not autosomal dominant |
|  | Chr9(GRCh37):g.117827190G>A  NM_002160.4:c.3223C>T  p.(Pro1075Ser) | Different from phenotype reported in the literature |
|  | Chr9(GRCh37):g.117852865C>A  NM_002160.4:c.433G>T  p.(Gly145Cys) | No phenotypic information available, subject not accessible |
| *TRRAP* | Chr7(GRCh37):g.98564703A>T  NM_003496.4:c.7141A>T  p.(Met2381Leu) | Different from phenotype reported in the literature |
| *USP48* | Chr1(GRCh37):g.22028059T>C  NM_032236.8:c.2659A>G  p.(Ser887Gly) | *In silico* prediction tools (MutationTaster update) |
| *WFS1* | Chr4(GRCh37):g.6293357A>G  NM_006005.3:c.631+263A>G  p.? | Different from phenotype reported in the literature |

^#^ One cell per subject.
