# Supplementary material for: Exome variant prioritization in a large cohort of hearing-impaired individuals indicates IKZF2 to be associated with non-syndromic hearing loss and guides future research of unsolved cases
Source: Hum Genet. 2024 Oct 16;143(11):1379–99. doi: 10.1007/s00439-024-02706-w (PMC11522133; doi:10.1007/s00439-024-02706-w)
Supplement: Supplementary file 13 — Supplementary file13 (DOCX 14 KB) [file 439_2024_2706_MOESM13_ESM.docx]

**Supplemental Table 10. Follow-up of selected variants in human deafness genes in group AD.**

| **Gene** | **Variant** | **Findings^#^** |
| --- | --- | --- |
| *MAP1B* | Chr5(GRCh37):g.71493033A>G  NM_005909.5:c.3851A>G  p.(Asn1284Ser) | Likely causative, no segregation analysis possible* |
| *PLS1* | Chr3(GRCh37):g.142408526T>G  NM_001145319.2:c.1048T>G  p.(Phe350Val) | Likely causative, no segregation analysis possible |
|  | Chr3(GRCh37):g.142422752G>A  NM_001145319.2:c.1414G>A  p.(Ala472Thr) | Patient is likely solved with other variant (*TRRAP*)* |
| *TJP2* | Chr9(GRCh37):g.71844171G>A  NM_004817.4:c.1520+5G>A  p.? | Likely causative, no segregation analysis possible* |
| *TNC* | Chr9(GRCh37):g.117853021C>T  NM_002160.4:c.277G>A  p.(Asp93Asn) | Variant does not co-segregate with HL |
|  | Chr9(GRCh37):g.117808955T>A  ENST00000341037.8_3.1:c.4313A>T  p.(Glu1438Val) | Variant does not co-segregate with HL |
| *TRRAP* | Chr7(GRCh37):g.98559074T>C  NM_001375524.1:c.6680T>C  p.(Met2227Thr) | Likely causative, no segregation analysis possible*  Gene was identified as human deafness gene after clinical exome sequencing |

* Two variants were identified in one subject (*MAP1B* and *TJP2* variants, based on literature there was no preference for one variant as causative to HL; *PLS1* and *TRRAP* variants, based on literature there was a preference for the *TRRAP* variant as causative to HL). ^#^ One cell per subject.
