# Supplementary material for: Exome variant prioritization in a large cohort of hearing-impaired individuals indicates IKZF2 to be associated with non-syndromic hearing loss and guides future research of unsolved cases
Source: Hum Genet. 2024 Oct 16;143(11):1379–99. doi: 10.1007/s00439-024-02706-w (PMC11522133; doi:10.1007/s00439-024-02706-w)
Supplement: Supplementary file 14 — Supplementary file14 (DOCX 13 KB) [file 439_2024_2706_MOESM14_ESM.docx]

**Supplemental Table 11. Flowchart of variant filtering in group AD, mouse deafness genes.**

| 27,089 variants | Selection: mouse deafness genes (orthologues of human deafness genes excluded)  Excluded: 26,302 variants |
| --- | --- |
| 787 variants | Selection: ≥3 samples  Excluded: 297 variants |
| 490 variants | Selection: subgroup of mouse deafness genes* (Supplemental Table 1)  Excluded: 457 variants |
| 33 variants | Selection: excluding artefacts (alignment files)  Excluded: 18 variants |
| Follow-up: 15 variants (Table 4) | |

List 2: mouse deafness genes. * Subgroup of mouse deafness genes that was used in the analysis of group AD based on personal communication and literature.
