# Supplementary material for: Exome variant prioritization in a large cohort of hearing-impaired individuals indicates IKZF2 to be associated with non-syndromic hearing loss and guides future research of unsolved cases
Source: Hum Genet. 2024 Oct 16;143(11):1379–99. doi: 10.1007/s00439-024-02706-w (PMC11522133; doi:10.1007/s00439-024-02706-w)
Supplement: Supplementary file 15 — Supplementary file15 (DOCX 15 KB) [file 439_2024_2706_MOESM15_ESM.docx]

**Supplemental Table 12. Follow-up of selected variants in orthologs of mouse deafness genes in group AD.**

| **Gene** | **Variant** | **Findings^#^** |
| --- | --- | --- |
| *CAMSAP3* | Chr19(GRCh37):g.7670336G>C  NM_020902.2:c.373G>C  p.(Ala125Pro) | Variant could not be validated by PCR and Sanger sequencing |
| *CELSR1* | Chr22(GRCh37):g.46931630G>A  NM_001378328.1:c.1438C>T  p.(Arg480*) | Variant does not co-segregate with HL |
|  | Chr22(GRCh37):g.46931039C>A  NM_001378328.1:c.2029G>T  p.(Val677Leu) | Variant does not co-segregate with HL |
|  | Chr22(GRCh37):g.46782296T>C  NM_001378328.1:c.6739+3A>G  p.? | No segregation analysis possible |
|  | Chr22(GRCh37):g.46762530C>A  NM_001378328.1:c.8206-153G>T  p.? | No segregation analysis because no strong candidate (not causative in other families) |
|  | Chr22(GRCh37):g.46759968G>A  NM_001378328.1:c.8960C>T  p.(Pro2987Leu) | Case was solved after inclusion |
| *IKZF2* | Chr2(GRCh37):g.213914526T>G  NM_001387220.1:c.485A>C  p.(His162Pro) | Candidate gene for further assessment |
|  | Chr2(GRCh37):g.213914502C>T  NM_001387220.1:c.509G>A  p.(Cys170Tyr) | Candidate gene for further assessment |
| *MAP3K1* | Chr5(GRCh37): g.56168693G>A  NM_005921.2:c.1547G>A  p.(Arg516Lys) | Variant could not be validated by PCR and Sanger sequencing |
|  | Chr5(GRCh37):g.56168810A>T  NM_005921.2:c.1664A>T  p.(Asp555Val) | Variant does not co-segregate with HL |
|  | Chr5(GRCh37):g.56177627A>C  NM_005921.2:c.2600A>C  p.(Glu867Ala) | Variant does not co-segregate with HL |
|  | Chr5(GRCh37):g.56177969C>T  NM_005921.2:c.2942C>T  p.(Pro981Leu) | No segregation analysis because no strong candidate (not causative in other families) |
| *USP42* | Chr7(GRCh37):g.6187470A>C  NM_001389650.1:c.1333A>C  p.(Lys445Gln) | Variant does not co-segregate with HL |
|  | Chr7(GRCh37):g.6155149A>G  NM_001389650.1:c.437A>G  p.(Lys146Arg) | Case was solved after inclusion |
|  | Chr7(GRCh37): g.6194334A>G  NM_001389650.1:c.3149A>G  p.(Tyr1050Cys) | Variant does not co-segregate with HL |

^#^ One cell per subject.
