# Supplementary material for: Exome variant prioritization in a large cohort of hearing-impaired individuals indicates IKZF2 to be associated with non-syndromic hearing loss and guides future research of unsolved cases
Source: Hum Genet. 2024 Oct 16;143(11):1379–99. doi: 10.1007/s00439-024-02706-w (PMC11522133; doi:10.1007/s00439-024-02706-w)
Supplement: Supplementary file 16 — Supplementary file16 (DOCX 19 KB) [file 439_2024_2706_MOESM16_ESM.docx]

**Supplemental Table 13.** **Air conduction thresholds of all subjects with an *IKZF2* variant.**

| **Family** | **Subject** | **Sex** | **Age at examination (y)** | **Source** | **Hearing thresholds (dB HL) for frequencies (kHz)** | | | | | | |
| --- | --- | --- | --- | --- | --- | --- | --- | --- | --- | --- | --- |
|  |  |  |  |  | **0.25** | **0.5** | **1** | **2** | **4** | **8** | **PTA** |
| W16-0482 | II:2 | Male | 56 | R | 30 | 35 | 55 | 105 | 105 | 95 | 75 |
|  |  |  |  | L | 30 | 45 | 75 | 95 | 105 | 90 | 80 |
|  |  |  |  | P95 | 13 | 14 | 17 | 21 | 31 | 44 | 21 |
|  | III:2 | Female | 26 | R | 15 | 20 | 45 | 45 | 50 | 70 | 40 |
|  |  |  |  | L | 15 | 20 | 50 | 40 | 40 | 65 | 38 |
|  |  |  |  | P95 | 7 | 8 | 8 | 8 | 9 | 10 | 8 |
| W22-1907 | II:3 | Female | 62 | R | 20 | 45 | 80 | 75 | 85 | 110 | 71 |
|  |  |  |  | L | 15 | 55 | 70 | 75 | 80 | 105 | 70 |
|  |  |  |  | P95 | 15 | 16 | 19 | 24 | 33 | 46 | 23 |
|  | II:4 | Male | 53 | R | 15 | 35 | 55 | 60 | 65 | 90 | 54 |
|  |  |  |  | L | 15 | 30 | 50 | 50 | 50 | 65 | 45 |
|  |  |  |  | P95 | 12 | 13 | 14 | 19 | 26 | 37 | 18 |
|  | III:1 | Male | 25 | R | 110 | 110 | 120 | 120 | 120 | 110 | 118 |
|  |  |  |  | L | 35 | 40 | 25 | 25 | 25 | 45 | 29 |
|  |  |  |  | P95 | 7 | 7 | 8 | 8 | 9 | 10 | 8 |
|  | III:2 | Female | 30 | R | 30 | 45 | 40 | 5 | 30 | 80 | 30 |
|  |  |  |  | L | 20 | 40 | 35 | 15 | 30 | 80 | 30 |
|  |  |  |  | P95 | 8 | 8 | 9 | 9 | 9 | 11 | 9 |
|  | III:3 | Male | 34 | R | 20 | 20 | 35 | 40 | 40 | 45 | 34 |
|  |  |  |  | L | 10 | 15 | 25 | 35 | 45 | 55 | 30 |
|  |  |  |  | P95 | 7 | 7 | 7 | 9 | 10 | 13 | 8 |
| W22-2757 | II:2 | Male | 59 | R |  | 15 | 25 | 55 | 55 | 95 | 38 |
|  |  |  |  | L |  | 30 | 30 | 60 | 60 | 90 | 45 |
|  |  |  |  | P95 | 14 | 16 | 18 | 24 | 36 | 50 | 24 |
|  | III:1 | Female | 40 | R | 15 | 30 | 50 | 40 | 25 | 60 | 36 |
|  |  |  |  | L | 20 | 25 | 50 | 45 | 40 | 65 | 40 |
|  |  |  |  | P95 | 15 | 30 | 50 | 40 | 25 | 60 | 15 |
|  | III:2 | Female | 36 | R | 5 | 5 | 45 | 60 | 55 | 70 | 41 |
|  |  |  |  | L | 0 | 0 | 40 | 55 | 50 | 60 | 36 |
|  |  |  |  | P95 | 9 | 9 | 8 | 8 | 10 | 13 | 9 |

Air conduction thresholds in dB hearing level (dB HL) of 0.25 to 8 kHz and the age- and gender-specific 95th percentile (P95, ISO 7029:2017 (International Organization for Stardardization 2017)) of all subjects with an *IKZF2* variant. dB HL, decibel hearing level; kHz, kilohertz; L, left ear; R, right ear, y, years.
