# Supplementary material for: Exome variant prioritization in a large cohort of hearing-impaired individuals indicates IKZF2 to be associated with non-syndromic hearing loss and guides future research of unsolved cases
Source: Hum Genet. 2024 Oct 16;143(11):1379–99. doi: 10.1007/s00439-024-02706-w (PMC11522133; doi:10.1007/s00439-024-02706-w)
Supplement: Supplementary file 17 — Supplementary file17 (DOCX 13 KB) [file 439_2024_2706_MOESM17_ESM.docx]

**Supplemental Results. Group AD, known human deafness genes.**

Variants in *MAP1B* (ACMG classification ‘unknown significance’: PM2, BP4), *TJP2* (ACMG classification ‘unknown significance’: PM2) and *TRRAP* (ACMG classification ‘unknown significance’: PM2) were considered as a likely cause of HL in the corresponding subjects. The variants in *MAP1B* and *TJP2* were found in one index case with mild-to-moderate HL (Supplemental Figure 2A) and an age of onset between 31 and 50 years. No family members were available for segregation analysis. For the *TRRAP* variant, segregation analysis was not possible because the subject was not receptive to further research within his/her family. *MAP1B* and *TRRAP* were not included in the gene panel for hearing impairment at the time of clinical exome sequencing. Two variants in *PLS1* were identified in two samples, one of which in the same sample as the *TRRAP* variant. This *PLS1* variant was not considered causative of the HL since the subject’s phenotype was more consistent with the reported DFNA75 (*TRRAP*, OMIM 618778) phenotype than with that of DFNA76 (*PLS1*, OMIM 602734) phenotype. This subject had mild HL (Supplemental Figure 2A) and an age of onset of 33 years. The second *PLS1* variant (ACMG classification ‘unknown significance’: PM2, PP3) was identified in a subject in whom also a variant in a candidate gene (*CELSR1*, Table 4) was identified. Also this subject was not receptive to confirm co-segregation of the variant with the HL in his/her family and no audiometric data were available. Finally, two variants in *TNC* were identified in two samples. These variants did not co-segregate with HL in either family.
